# Protein Thermostability Is Owing to Their Preferences to Non-Polar Smaller Volume Amino Acids, Variations in Residual Physico-Chemical Properties and More Salt-Bridges

**DOI:** 10.1371/journal.pone.0131495

**Published:** 2015-07-15

**Authors:** Anindya Sundar Panja, Bidyut Bandopadhyay, Smarajit Maiti

**Affiliations:** 1 Post Graduate Department of Biotechnology, Oriental Institute of Science and Technology, Vidyasagar University, Midnapore, 721102, West Bengal, India; 2 Post Graduate Department of Biochemistry and Biotechnology, Cell and Molecular Therapeutics Laboratory, Oriental Institute of Science and Technology, Vidyasagar University, Midnapore, 721102, West Bengal, India; Nanyang Technological University, SINGAPORE

## Abstract

**Introduction:**

Protein thermostability is an important field for its evolutionary perspective of mesophilic versus thermophilic relationship and for its industrial/ therapeutic applications.

**Methods:**

Presently, a total 400 (200 thermophilic and 200 mesophilic homologue) proteins were studied utilizing several software/databases to evaluate their amino acid preferences. Randomly selected 50 homologous proteins with available PDB-structure of each group were explored for the understanding of the protein charges, isoelectric-points, hydrophilicity, hydrophobicity, tyrosine phosphorylation and salt-bridge occurrences. These 100 proteins were further probed to generate Ramachandran plot/data for the gross secondary structure prediction in and comparison between the thermophilic and mesophilic proteins.

**Results:**

Present results strongly suggest that nonpolar smaller volume amino acids Ala (*χ* ^2^ = 238.54, p<0.001) and Gly (*χ* ^2^ = 73.35, p<0.001) are highly and Val moderately (*χ* ^2^ = 144.43, p<0.001) occurring in the 85% of thermophilic proteins. Phospho-regulated Tyr and redox-sensitive Cys are also moderately distributed (*χ* ^2^~20.0, p<0.01) in a larger number of thermophilic proteins. A consistent lower distribution of thermophilicity and discretely higher distribution of hydrophobicity is noticed in a large number of thermophilic versus their mesophilic protein homolog. The mean differences of isoelectric points and charges are found to be significantly less (7.11 vs. 6.39, p<0.05 and 1 vs. -0.6, p<0.01, respectively) in thermophilic proteins compared to their mesophilic counterpart. The possible sites for Tyr phosphorylation are noticed to be 25% higher (p<0.05) in thermophilic proteins. The 60% thermophiles are found with higher number of salt bridges in this study. The average percentage of salt-bridge of thermophiles is found to be higher by 20% than their mesophilic homologue. The GLU-HIS and GLU-LYS salt-bridge dyads are calculated to be significantly higher (p<0.05 and p<0.001, respectively) in thermophilic and GLU-ARG is higher in the mesophilic proteins. The Ramachandran plot/ data suggest a higher abundance of the helix, left-handed helix, sheet, nonplanar peptide and lower occurrence of *cis* peptide, loop/ turn and outlier in thermophiles. Pearson’s correlation result suggests that the isoelectric points of mesophilic and thermophilic proteins are positively correlated (r = 0.93 and 0.84, respectively; p<0.001) to their corresponding charges. And their hydrophilicity is negatively associated with the corresponding hydrophobicity (r = -0.493, p<0.001 and r = -0.324, p<0.05) suggesting their reciprocal evolvement.

**Conclusions:**

Present results for the first time with this large amount of datasets and multiple contributing factors suggest the greater occurrence of hydrophobicity, salt-bridges and smaller volume nonpolar residues (Gly, Ala and Val) and lesser occurrence of bulky polar residues in the thermophilic proteins. A more stoichiometric relationship amongst these factors minimized the hindrance due to side chain burial and increased compactness and secondary structural stability in thermophilic proteins.

## Introduction

Discovery of the bacterium *Thermus aquaticus* initiates a significant and active research on the thermostable organisms [1]. These organisms are optimally grown in the range of 45–80°C temperature, whereas; this range of the mesophilic organisms is 15–45°C [2,3]. Thermophiles include eubacteria, archaea and some fungi. These are more phylogenetically diverse and extensively evolutionized [2,3]. The phenotypic characteristics of thermophilicity of an organism are mainly conferred by its metabolic integrity at higher temperature. This integrity is attributed by the protein thermostability of the organism [4]. The protein is the most dependable and inheritable molecular machines which take an important part in the adaptation process. Its function is defined by its amino acid sequence and structural identity [5]. Environmental stress is the main driving force for the adaptation. The natural selection pressure is the key regulator for the adaptation and evolution mechanism. It influences the phenotype characteristic of the organism by shaping the genotypes modifications which are practically exhibited in the form of qualitative and quantitative changes in their protein characteristics [6,7].

The increased thermostability of the protein is attributed by its higher hydrophobicity and compactness [8], greater polar surface area, examined in 16 families of proteins [9], smaller surface-area to volume ratio and fewer thermolabile residues, explored in D-glyceraldehyde-3-phosphate dehydrogenase (GAPDH) from the extreme thermophile *Thermus aquaticus* [10]. The structural resilience and the dynamic nature of a protein macromolecule attribute to its global thermal adaptation as concluded from the studies on the hyperthermophile malate dehydrogenase from *Methanococcus jannaschii* and a mesophile, the lactate dehydrogenase from *Oryctolagus cunniculus* [11]. A significant decrease in the frequency of glutamine is noticed in thermophiles [12]. At the gene level, the extrinsic selective force is found to be linked to the process of synonymous codon usage for some amino acids particularly for the arginine and isoleucine in thermophiles. It is reasonable to assume that the higher GC content in the DNA is an important contributing factor for genome stability, which has been studied by the hierarchical clustering from the genomic sequences of six thermophilic archaea, two thermophilic bacteria, 17 mesophilic bacteria and two eukaryotic species [13]. But, it is also evident that the global amino acid composition alone (irrespective of the DNA composition) may be a dependable factor determining protein thermostability [13]. One report reveals a similar rate of occurrence of polar, nonpolar amino acids and compactness in thermophilic and mesophilic proteins [14]. Thermophilic proteins are more resistant to proteolysis and chemical denaturation; hence there is an interest in engineering hyperstable biocatalysts adopting the same mechanism that nature opts [2,3]. Thermophilic polymerases, proteases, amylases and xylanases already have industrial applications [15,16]. Most of the previous studies dealt with a smaller number of proteins and lesser number of possible thermostabilizing factors in single dataset [9,16]. Some of those utilized the purified or cloned-purified single thermostable proteins from a specific or model organism [10,11]. In 2011, Sawle and Ghosh investigated on a dataset of 116 proteins (largest in that period) to explore mainly the thermodynamic basis of protein thermostability [17]. In 2012, Meruelo *et al*. explored the variations between the thermophilic and mesophilic membrane proteins (25 and 101, respectively) [18]. An extensive genome-wide study with a large number of orthologous genes from archaea and bacteria revealed that the synonymous or non-synonymous nucleotide substitution is very lower in thermophiles than the non-thermophiles [19]. In that study, the investigator hinted on the influences of the natural selection and species’ environment on the thermophilic protein stabilization/adaptation [19]. Studies on these aspects, utilizing more interactive analysis of multiple stabilizing factors in a comparatively large number of proteins are inadequately focused in several earlier investigations.

In this study, for the first time with a large homolog protein dataset (total 400), we broadened our investigation to elucidate the major contributing factors encompassing the protein thermostability. Two hundred thermophilic and their homolog 200 mesophilic proteins were selected to delineate the pattern of the amino acids occurrence and preferences. Randomly selected fifty proteins from each group were studied to investigate their physicochemical behaviors (viz. hydrophilicity, hydrophobicity, charge and isoelectric point). The rate of occurrence of the salt bridges and properties defined for protein Ramachandran plot/data was elucidated. The rate of Tyr phosphorylation was also studied with these 100 proteins. A large number of analytical methods have been employed here and several statistical analyses were utilized to explore the possible association amongst different thermo-stabilizing factors. The present results are important and discussed/ analyzed to explain the global protein thermostability.

## Materials and Methods

### Determination of the occurrence rate and preferences of amino acids in thermophilic and mesophilic proteins

The database of the website http://www.uniprot.org/ was utilized for finding the amino acid sequences of thermophilic and mesophilic proteins [20]. Two hundred thermophilic and their homologous two hundred mesophilic proteins (Table A in S1 File) were downloaded and the percentage of 20 amino acids in each protein was calculated by accessing the website http://www.ebi.ac.uk/Tools/services/Pepstat [21]. The frequency zone was arranged in ascending order. Here, the Sturges formula (k = 1+3.322 log_10_ N) was used to find the class interval and desirable number of groups into which the distribution of observations was classified.

Maximum value of different amino acids (Mx) was 20 and the minimum value (Mn) was 0. Total number of observations (N) in each group was 200. The range (Rx) would be (20–0) = 20. So, the number of class interval (k) = 1+3.322 log_10_ N = 1+3.322 log_10_200 = 8.644 ~ 9. Now, h = Rx/k, where, h = size of the class intervals, Rx = range = 20, k = class interval = 9. So, the value of h would be = 20/9 = 2.22 ~ 2.00. The occurrences of 200 thermophilic and their 200 mesophilic protein homologues (y-axis) were categorized with respect to the percentage of the abundance of particular amino acids (0–20% on x-axis) and plotted as a bar-line plot/diagram [22]. The graph represents a comparative assessment of amino acid abundance between two different types of proteins.

For the analysis of amino acid sequence of the heat shock proteins, the ExPASy (Expert Protein Analysis System) proteomics server of the Swiss Institute of Bioinformatics (SIB) was utilized. To calculate the amino acid ratio present in proteins or enzymes, we used http://pir.georgetown.edu/ database [23]. The amino acid sequence of a particular protein was statistically calculated by using the molecular composition programme present in the pir database.

### Assessment of the physico-chemical behavior of thermophilic and mesophilic proteins

To study the hydrophilicity, hydrophobicity, isoelectric-point and charge characters, fifty thermophilic (Table B in S1 File) and fifty of their homologous mesophilic proteins (Table C in S1 File) were randomly selected out of early mentioned 200 proteins from each group (Table A in S1 File). The percentages of the hydrophobic (M, F, A, I, L, V, W and P), hydrophilic (K, R, D, E and H) and other residues (S, G, C, T, N, Q and Y) were computed. The backbone conformations of both types of proteins were verified by the Peptide Property Calculator server https://www.genscript.com/ssl-bin/site2/peptide_calculation.cgi for studying the above mentioned physico-chemical characters [24]. The occurrences of 50 thermophilic and 50 mesophilic protein homologues (y-axis) were categorized with respect to their values of charge and isoelectric point and percentage of hydrophilicity and hydrophobicity (x-axis). These were plotted as the line diagram.

### Assessment of salt-bridges in thermophilic and mesophilic proteins

To study the occurrence rate of the salt bridges, fifty thermophilic and their homolog fifty mesophilic proteins (Table B and C in S1 File) which has their resolved 3D structure in PDB and has been utilized in the evaluation of physico-chemical properties) were selected. The salt-bridge analysis tool of Visual Molecular Dynamics, VMD (http://www.ks.uiuc.edu) was utilized for this study [25]. VMD can read standard Protein Data Bank (PDB) files and display as the requirements were fed. VMD was utilized in this study to animate and analyze the trajectory of a molecular dynamics (MD) simulation. The number of total salt-bridges was calculated and their percentage was derived with respect to the total number of residues in the corresponding proteins. The abundance rates of different important salt bridge dyads (ASP-ARG, ASP-HIS, ASP-LYS, GLU-ARG, GLU-HIS, GLU-LYS) were determined and calculated as the percentage of total number of salt bridges in the corresponding proteins [26].

### Visualization of surface and core salt bridges

Further, to analyze the nature and position of the salt bridges of different thermophilic and mesophilic proteins, we have used the software RasMol 2.7.5 a Molecular Graphics Visualization Tool with command line option and visualized the nature of these salt bridges as per the positions of the amino acid residues of the selected thermophilic (5) and mesophilic (5) proteins [27].

### Evaluation of tyrosine-phosphorylation in thermophilic and mesophilic proteins

The 50 thermophilic and 50 homolog mesophilic proteins (used in physico-chemical properties and total salt-bridge studies, Table B and C in S1 File) were evaluated with different ranges (0–5, 5–10, 10–15 and 15–20) for Tyr phosphorylation utilizing the online analysis tools (http://www.geneinfinity.org/sp/sp_proteinptmodifs.html) and the Group-based Prediction System, (GPS ver 2.0); http://gps.biocuckoo.org [28,29]. The average occurrence (in the two groups of these 100 proteins) of Tyr-phosphorylation/ 100 amino acid residues was calculated as mean ± SE and compared.

### Ramachandran plot data were generated for 50 thermophilic and 50 mesophilic proteins

The proteins (Table B and C in S1 File) which were used for the assessment of the salt bridges and other physico-chemical properties, further utilized to generate the Ramachandran plot utilizing the STAN—the STructure ANalysis server (Uppsala Software Factory) [30]. The facilities were provided to use the Software and Resources for Macromolecular Crystallography and Structural Biology to develop Ramachandran Plot data from protein structure deposited in the PDB. This service was based on the Moleman2 program developed by Gerard Kleywegt [31]. The resolved PDB structure of a total 50 thermophilic proteins and their homolog 50 mesophilic proteins were utilized to generate the Ramachandran plot in 5 windows (10 proteins x 5) for each type (thermophilic and mesophilic) of protein. The logistic values originated during the plot generation were presented in a table.

### Statistical analysis

The statistical analyses were done by using the SPSS for Windows statistical software package (SPSS Inc., Chicago, IL, USA, 2001). The student-'t' test was employed to evaluate the differences of means of several salt-bridges groups within or between thermophilic and mesophilic proteins. Comparisons were analyzed by Pearson's *χ* ^2^ test between baseline categorical-variables like protein/ amino acid types and residual distribution/ preference-outcome. The Pearson correlation (considered significant at a level p<0.05) was employed for assessing the continuous dependent-variables (isoelectric point, charge, hydrophilic, hydrophobic and salt-bridges etc).

## Results

**Fig 1:**
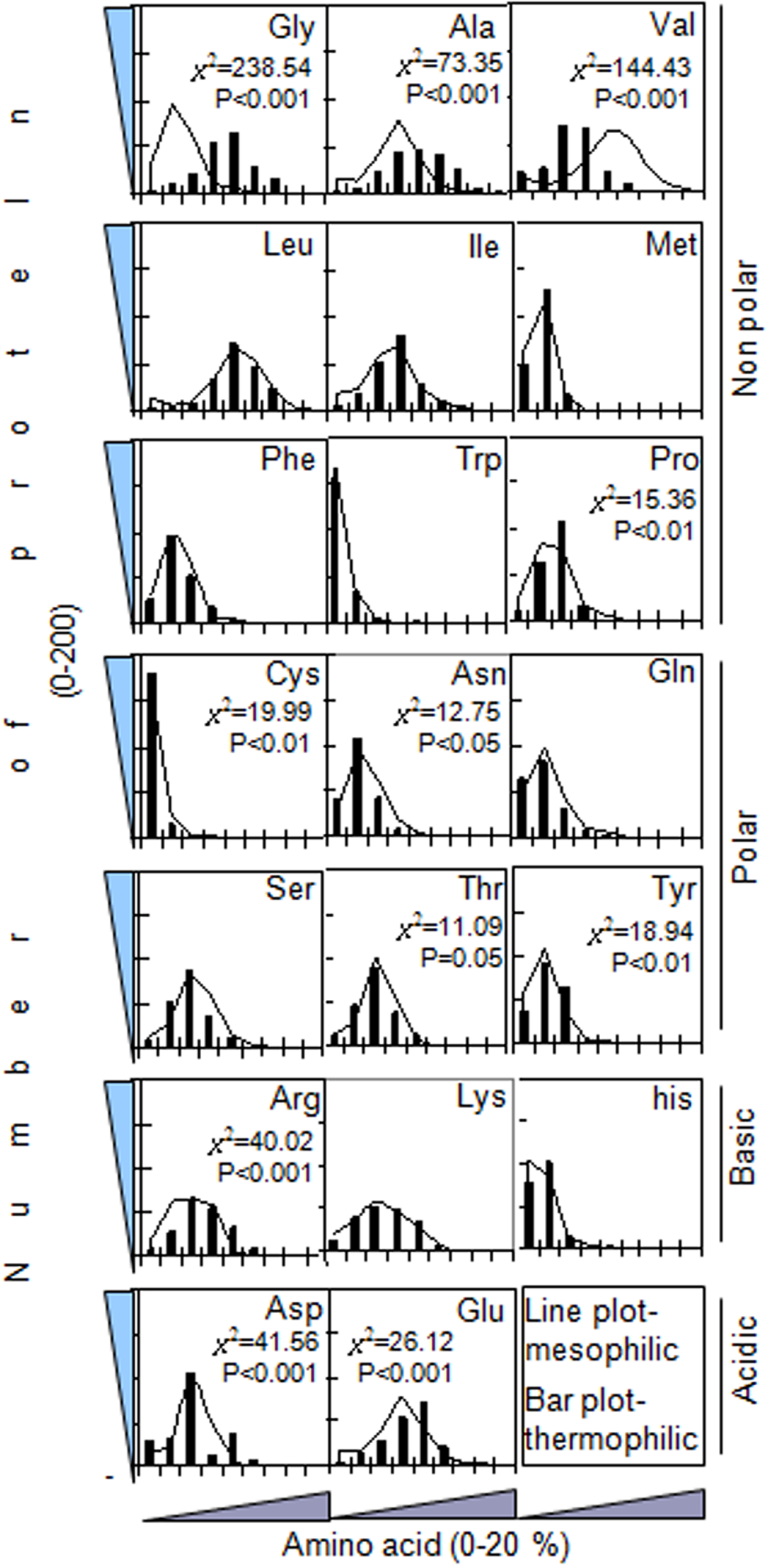
Amino acid distributions are shown in 200 mesophilic and their 200 thermophilic homologue proteins. The database from the website http://www.uniprot.org/ was utilized for finding the amino acid sequences of thermophilic and mesophilic proteins. The percentage of 20 amino acids in each protein is calculated by accessing the website http://www.ebi.ac.uk/Tools/services/Pepstat. The occurrences of amino acid are represented as different groups like 0–2%, 2–4%.....up to 18–20%. The distribution pattern in thermophilic versus mesophilic proteins are further analyzed by the Chi-square test.

The relative abundance of 20 amino acids was determined in 200 mesophilic and their homolog 200 thermophilic proteins in the present study (Fig 1). The present result indicates any specific preferences of residues which are occurring at high, moderate or low level in two types of proteins. In the amino-acid distribution plot of Fig 1, the X-axis represents the ranges of occurring amino acids, i.e. 0–2%, 2–4%, 4–6% and so on. The Y-axis represents the number of proteins that comprise a certain % category of amino acids in their polypeptide chain.

It is noticed that when more than 85% of thermophilic proteins are constituted with 6–8% → 12–14% of glycine, 90% of mesophilic proteins carries 4–6% of this amino acid (*χ* ^2^ = 238.54, p<0.001). The results from Fig 1 suggest the higher rate of occurrence of Ala in a number of thermophilic proteins, but lower rate was occurring in more mesophilic proteins. Around 17% of thermophilic proteins contain more than 12% of Ala in their polypeptide chains; whereas, only 2% of mesophilic proteins carry this amount (*χ* ^2^ = 73.35, p<0.001). The Val is present with (0–10%) in 80–90% of thermophilic and more is present in a larger number of mesophilic proteins (*χ* ^2^ = 144.43, p<0.001). The Trp was similarly occurring (0–2 → 2–4%) in 95% of thermophilic and mesophilic proteins. The 50% of the mesophilic proteins showed a higher abundance of 0–2 → 2–4% of Pro than their homologue, whereas 50% of thermophilic proteins showed a higher abundance of 4–6% of this residue (*χ* ^2^ = 15.36, p<0.01).

A larger and similar fraction of both thermophilic and mesophilic proteins constitute 12–14% of leucine, 2–4% → 4–6% of Met, 8–10% of lysine. Major polar amino acids showed similar but lower rate of occurrence in both types of proteins except that Tyr (*χ* ^2^ = 18.94, p<0.01) and Asn (*χ* ^2^ = 12.75, 0.05) residues. Most of the thermophilic and mesophilic proteins (~90%) constitute very lower occurrence rate (0–4%) of Cys residues, but at 0–2% range, a little higher rate in thermophile (*χ* ^2^ = 19.99, p<0.01) is evident. The Ser and Thr are present with 6–8% in ~90% of the thermophilic and mesophilic proteins. In this study, a higher % of mesophilic proteins comprise 0–2 → 2–4% of arginine, whereas some thermophilic proteins contain 8–10 → 10–12% of this amino acid residue (*χ* ^2^ = 40.02, p<0.001). Moderately higher rate of distribution of Asp is evident in the case of a larger number of thermophilic proteins (*χ* ^2^ = 41.56, p<0.001). For the residue Glu, lower percentage (0–2 → 6–8%) is higher in mesophilic and a higher percentage (8–10 → 14–16%) is present in the thermophilic proteins (*χ* ^2^ = 26.12, p<0.001) (Fig 1).

**Fig 2:**
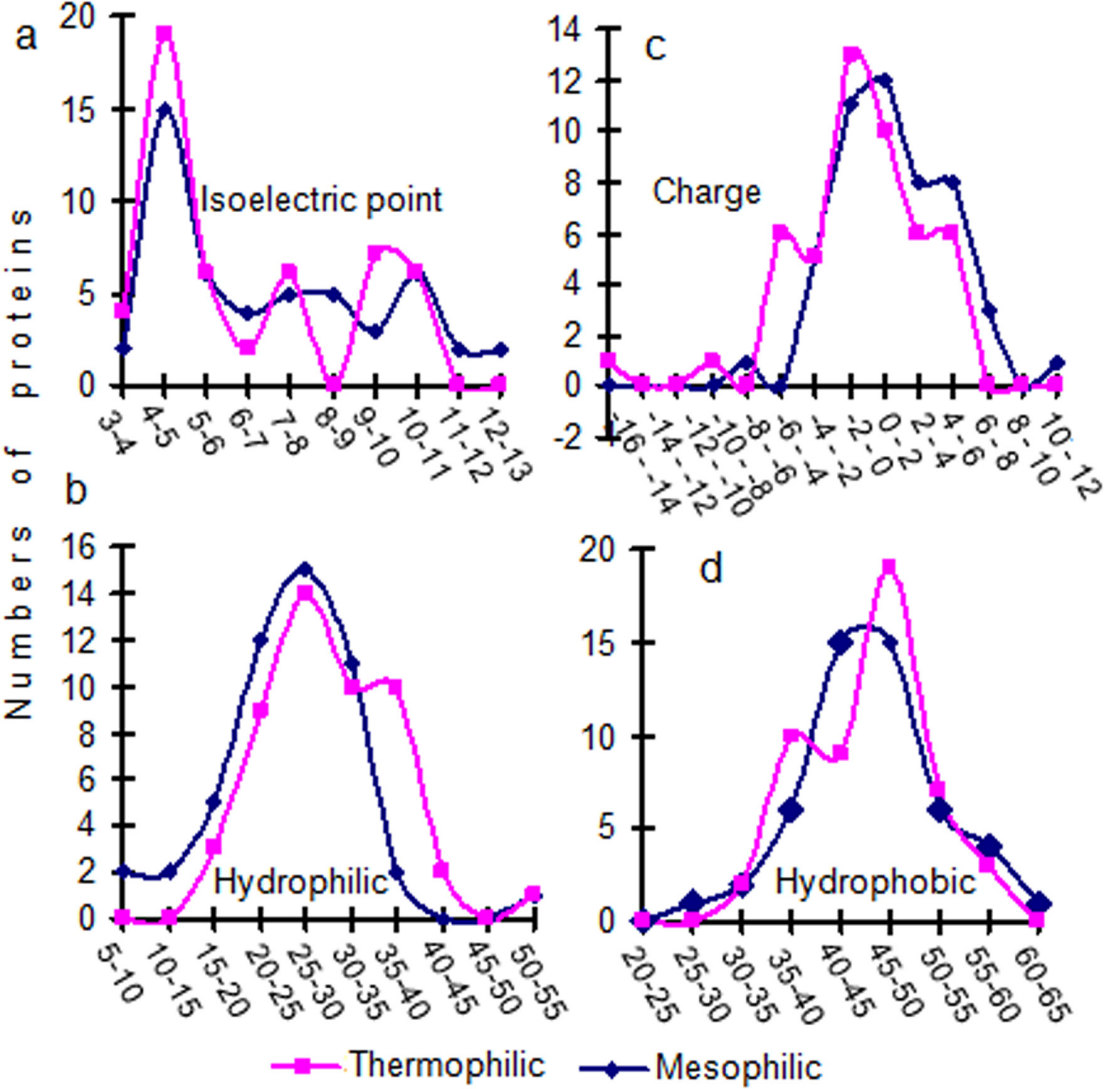
Distribution of 50 thermophilic and their homologue mesophilic proteins are shown in line plot according to their isoelectri points, charges, hydrophilicity and hydrophobicity. The backbone conformations of both types of proteins were verified by the Peptide Property Calculator server https://www.genscript.com/ssl-bin/site2/peptide_calculation.cgi. The values of these physico-chemical parameters are grouped in different categories according to their corresponding range and plotted in the lower axis.

**Fig 3:**
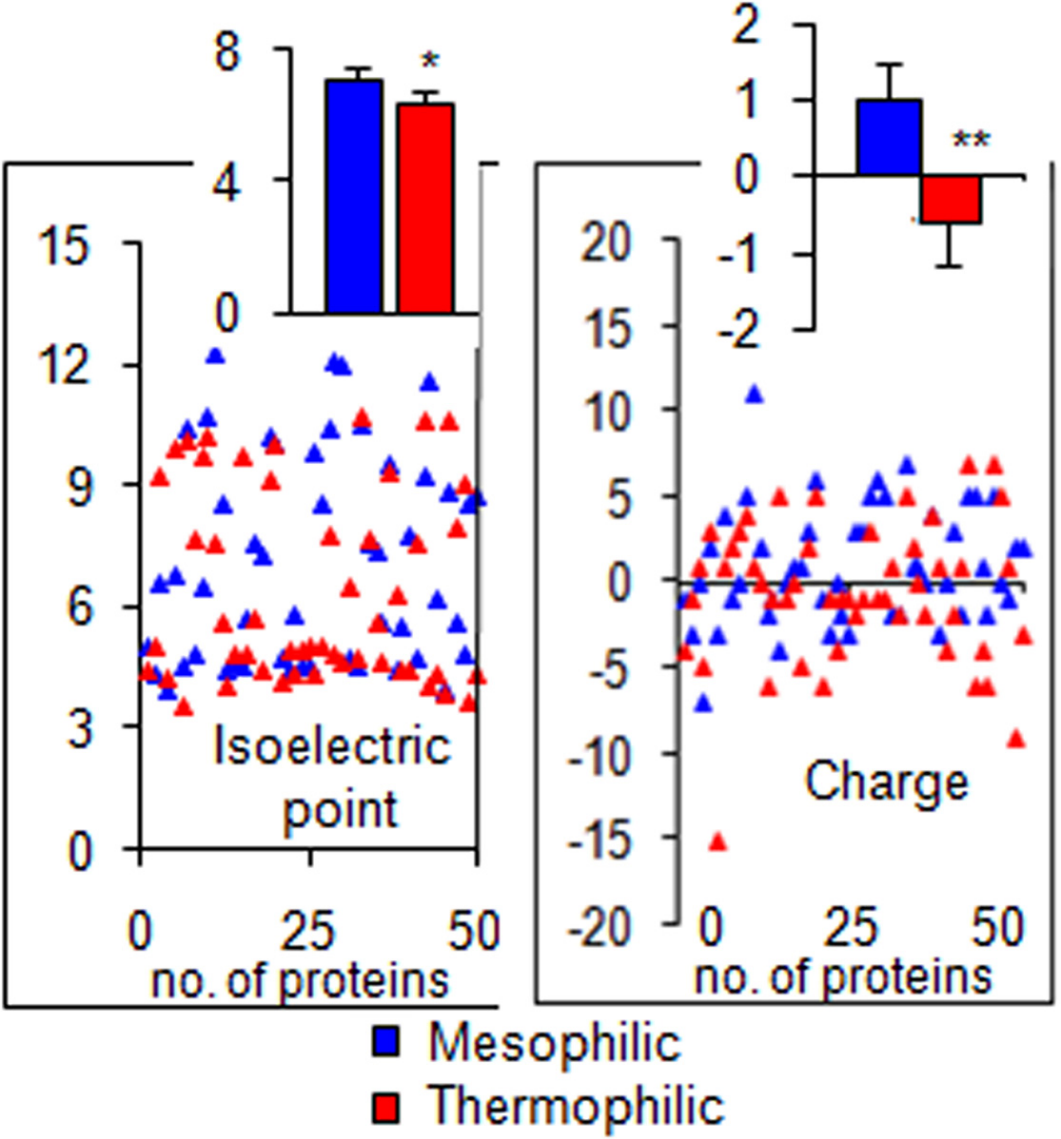
Distribution and deviations of individual values of isoelectric point and charge of 50 thermophilic and their homologue mesophilic proteins are shown in dot plot and the average values (mean ± SE) of those are plotted in the inset as bar diagram. The backbone conformations of both types of proteins were verified by the Peptide Property Calculator server https://www.genscript.com/ssl-bin/site2/peptide_calculation.cgi. The level of significances of the difference of mean are calculated by Student’s t test and represented on the bar as “*”. *p<0.05 and **p<0.01.

Present results from 50 thermophilic and 50 mesophilic proteins suggest that hydrophobicity in a larger number of thermophilic proteins was consistently higher than their homologous mesophiles (Fig 2). Hydrophilicity, up to a certain level was lower in a greater number of thermophilic proteins, but at higher levels it was comparatively higher in these proteins. The number of proteins is also shown to be differentially segregated according to the ranges of their isoelectric points and charges (Fig 2). The segregation of an individual value of isoelectric point and charges from each of 50 thermophilic and 50 mesophilic proteins are presented in Fig 3. And the mean and SE values of these parameters presented in the inset. These data show a significantly lower isoelectric point (p<0.05) and charge (p<0.01) in thermophilic proteins. When the average charge in mesophiles is found to be a positive value (at neutral pH), the same is found to be negative in thermophiles (Fig 3).

**Fig 4:**
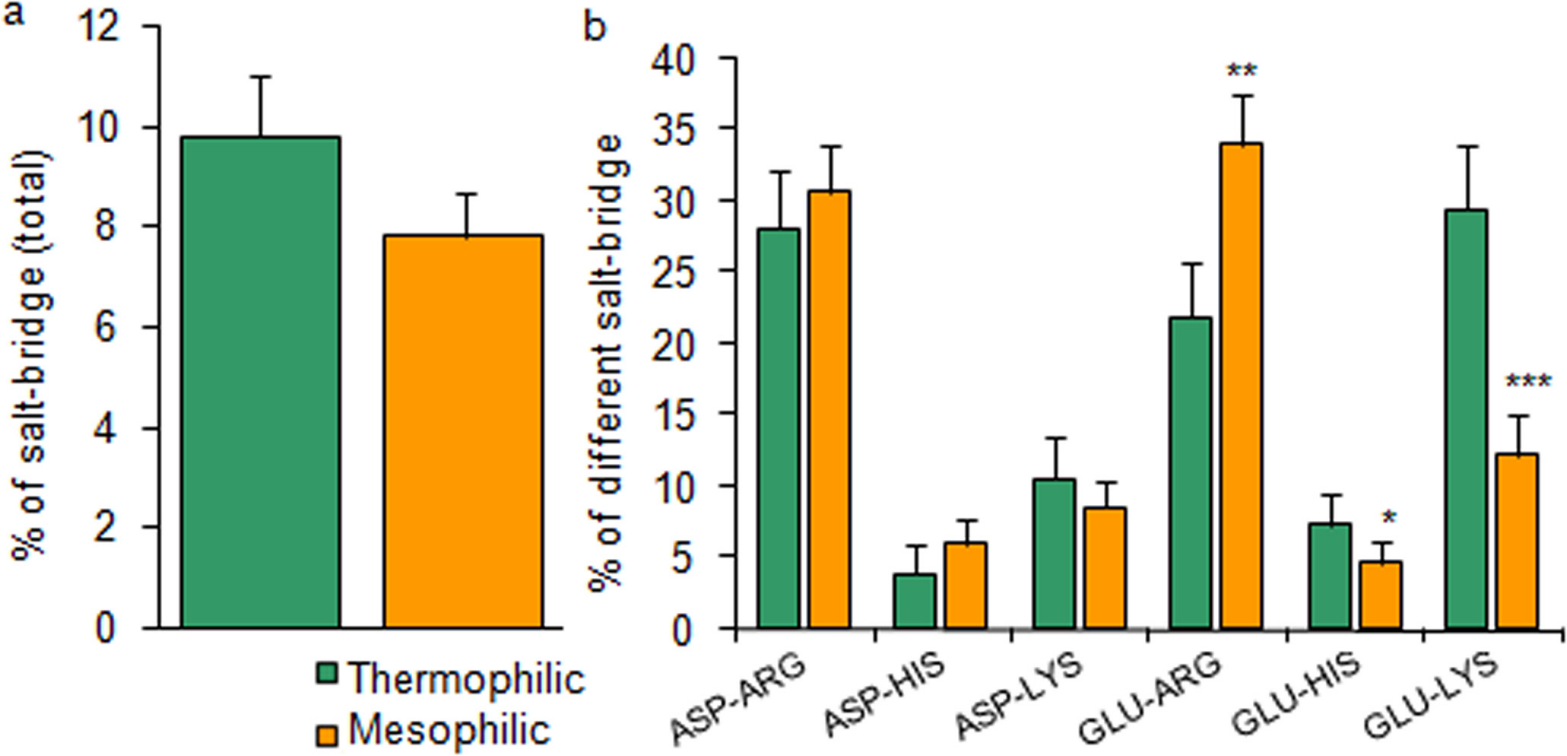
Fifty thermophilic and their homologue fifty mesophilic proteins (which has their resolved 3D structure in PDB and has been utilized in the evaluation of physic-chemical properties) were selected. The salt-bridge analysis tool of Visual Molecular Dynamics, VMD (http://www.ks.uiuc.edu) was utilized for this study. The number of total salt-bridges was calculated as the percentage derived with respect to the total number of residues in the corresponding proteins. The abundance rate of different important salt bridge dyads (ASP-ARG, ASP-HIS, ASP-LYS, GLU-ARG, GLU-HIS, GLU-LYS) were determined and calculated as the percentage of total number of salt bridges in those proteins. The level of significances of difference of mean are calculated by Student’s t test and represented on the bar as “*”. *p<0.05, **p<0.01 and ***p<0.001.

The present results suggest that 60% of the studied thermophilic proteins have a higher occurrence rate of salt bridges than their mesophilic counterpart and 40% mesophilic protein have a higher rate. The average number of salt bridge from studied total 50 thermophilic proteins is ~20% higher than that of 50 of their homolog mesophiles (Fig 4A). When the salt bridge GLU-HIS and GLU-LYS are significantly higher occurring (p<0.05 and p<0.001, respectively) in thermophilic proteins the GLU-ARG is higher in the mesophilic proteins than their corresponding homologue. Individual salt bridges were calculated as their number per hundred of total salt bridges in that protein (Fig 4B).

**Fig 5:**
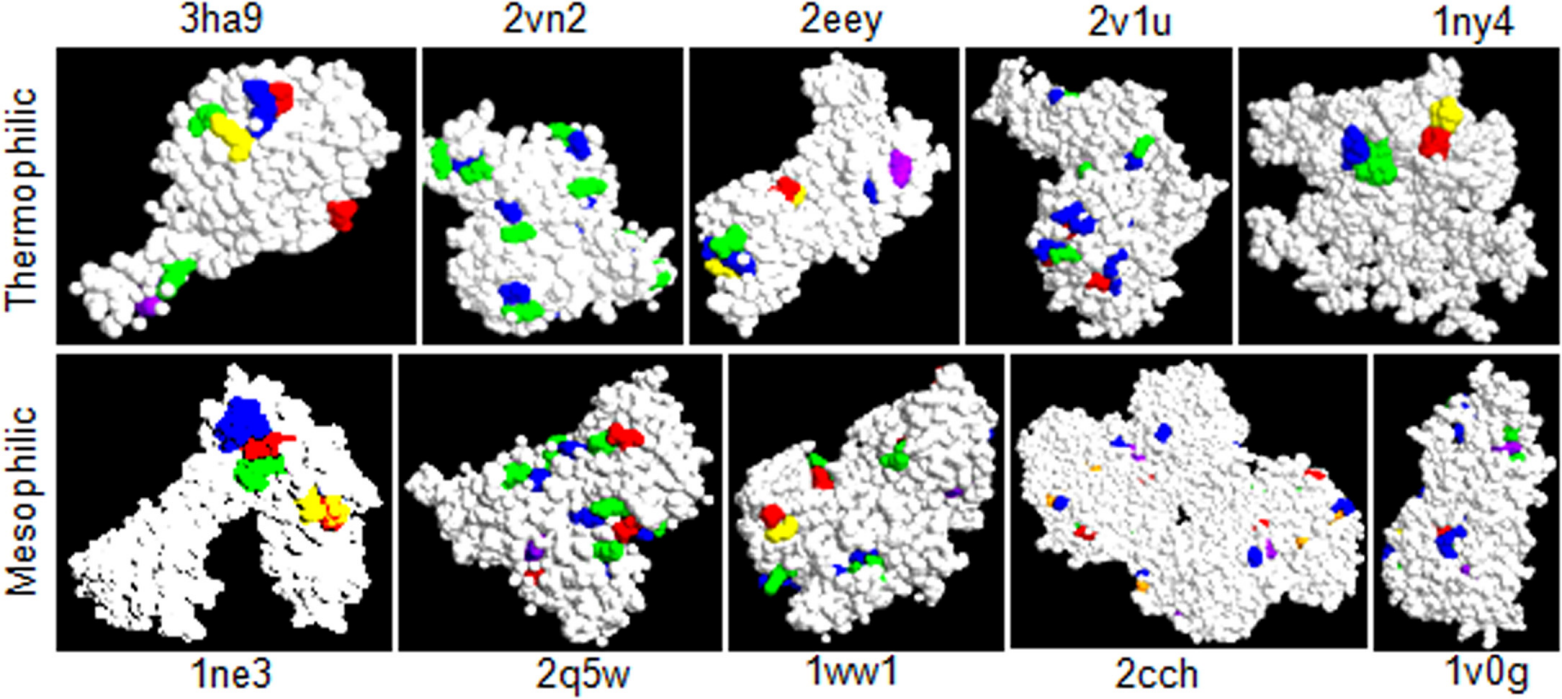
To analyze the nature and position of the salt bridges of different thermophilic and mesophilic proteins, we have used the software RasMol 2.7.5 a Molecular Graphics Visualisation Tool.

The Fig 5 depicts the RasMol visualization model showing apparent location of the surface/core salt bridges in thermophilic and homolog mesophilic proteins. The thermophilic proteins are found to be smaller in volume and the calculation suggests a ~ 9% lowering of the average residue number in the thermophilic proteins (50) with comparison to their mesophilic homolog (50). This may be apparent to say that number of core salt-bridges is found to be embedded in the mesophilic proteins.

**Fig 6:**
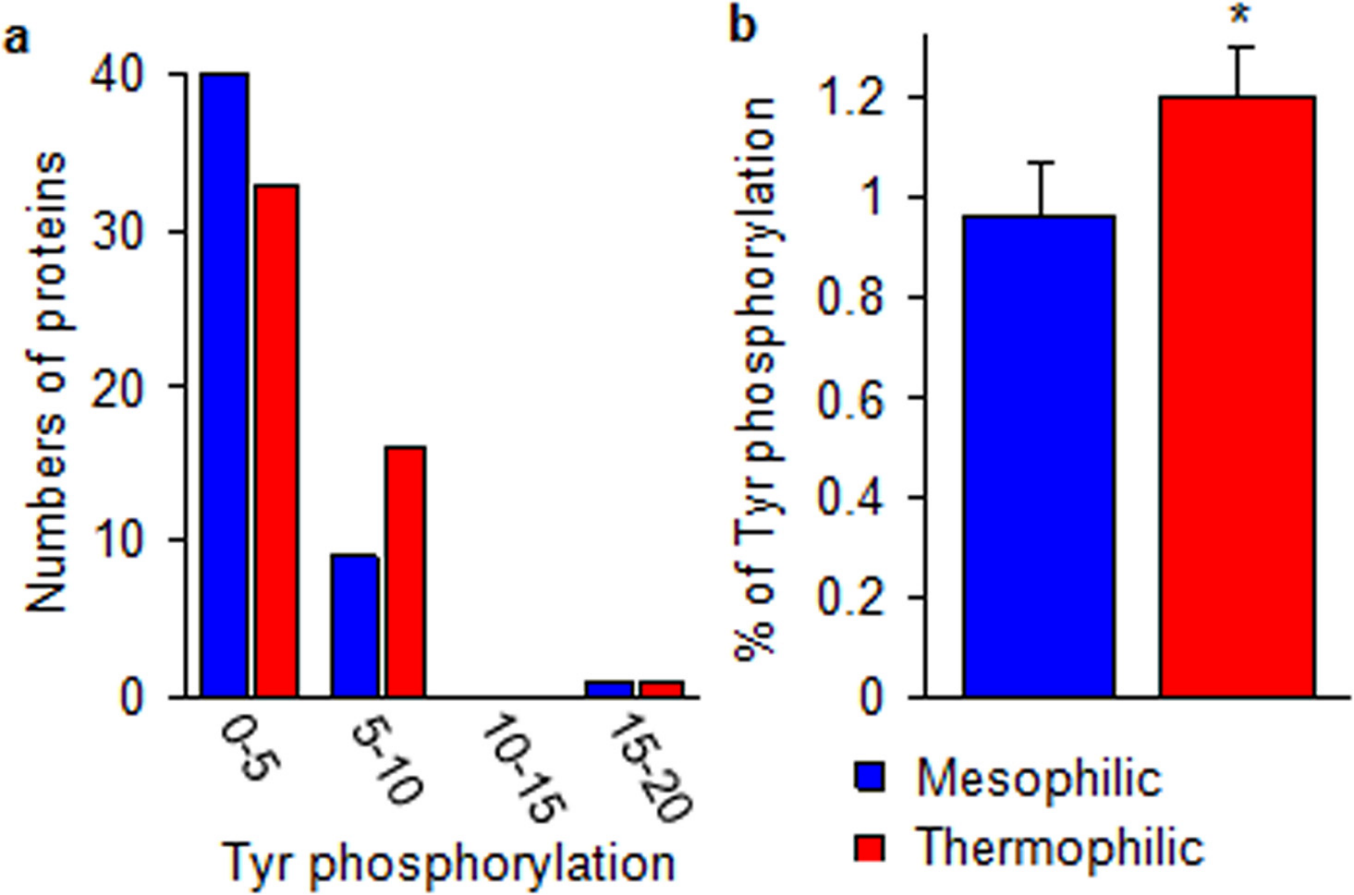
Tyrosine phosphorylation is presented. The Tyr phosphorylation is evaluated at different ranges and presented in fifty thermophilic and their homologue fifty mesophilic proteins (used in salt bridge and physicochemical property study) (a). The mean number (mean ± SE) of Tyr which undergoes phosphorylation was calculated (50 thermophilic and 50 mesophilic proteins) as the percentage derived with respect to the total number of residues in the corresponding proteins. Level of significance *p<0.05.

The Fig 6 shows the distribution of 50 thermophilic and 50 mesophilic proteins at different ranges (Tyr number as 0–5, 5–10, 10–15 and 15–20) of Tyr phosphorylation. At 0–5 range, 10% more mesophilic protein shows Tyr phosphorylation. But, at 5–10 range, 40% more thermophilic protein shows Tyr phosphorylation (a). The average occurrence of Tyr-phosphorylation per 100 amino acid residues of thermophilic proteins is found to be higher by 25% than their homolog mesophiles (p<0.05) (b). The higher level of phospho-modification of Tyr in thermophiles suggests enhanced metabolic regulations in this group.

**Fig 7:**
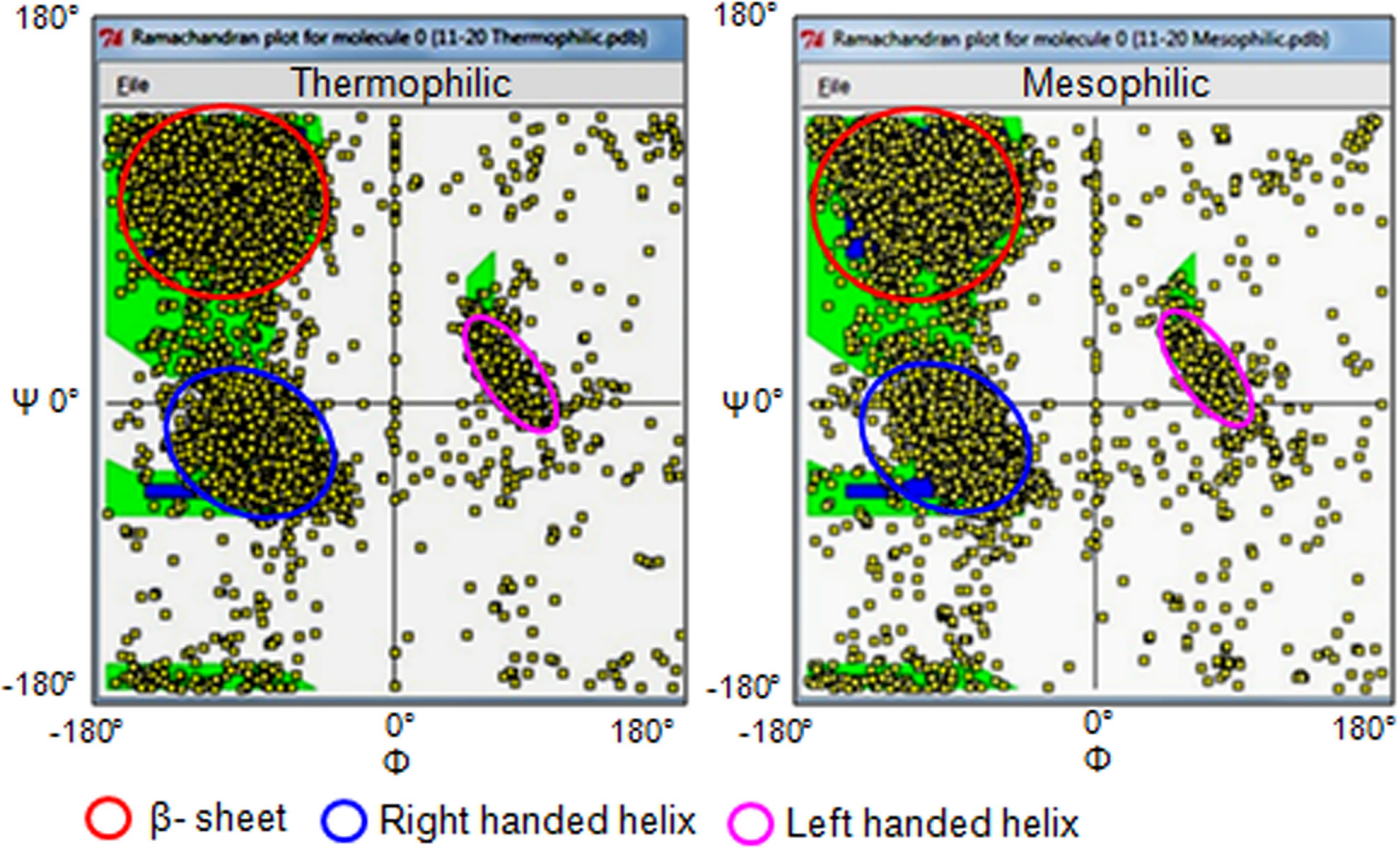
Representative Ramachandran-plot for 10 thermophilic proteins together (left panel) and their homologue 10 mesophilic proteins (right panel) are shown. The resolved PDB structure of a total 50 thermophilic proteins and their homologue total 50 mesophilic proteins were probed to generate the Ramachandran plot utilizing the STAN—the STructure ANalysis server (Uppsala Software Factory).

The details of the logistic outcome of the Ramachandran plot suggest that the average number of residues is higher in mesophilic proteins in comparison to their thermophilic counterpart (Table 1, Fig 7). The residues in core areas of β-sheet, right handed and left handed helix are found more condensed in the thermophilic protein. The residues in the outliers and disallowed regions are noticed to be abundant in the mesophilic protein. Taking into account, the smaller average residual number of thermophilic proteins, it is determined that the thermophilic proteins constitute with higher number of beta sheet, left or right handed helix (Table 1). Higher occurrence of glycine, pre-proline and proline is also predicted in the thermophilic proteins.

**Table 1:** The logistic representations of the Ramachandran plot data of thermophilic and mesophilic proteins. It shows marked variations in their secondary and higher structural features. The STAN—the STructure ANalysis server (Uppsala Software Factory) was utilized to generate these data. This service is based on the Moleman2 program developed by Gerard Kleywegt.

Ramachandran plot data	Thermophilic	Mesophilic
Total no of residues checked	32337	34270
% of residues in α- helix	44.155	42.089
% of residues in β- sheet	23.075	22.111
% of residues in loop or turn	10470 (32.38)	12151 (45.46)
% of residues in left handed α- helix	0.384	0.341
Cis-peptide bonds	74	113
Nonplanar peptide bonds	42	16
Glycine residues	2520 (7.79%)	2555 (7.45%)
Residues in core region	28251 (87.36%)	29950 (87.39%)
Average % of outlier from different chains	3.97; (2.3–4.9)	4.45; range (1.2–8.3)
Disallowed residues	716 (2.3%)	735 (2.1%)
No. residues of type non-protein	235	196

**Fig 8:**
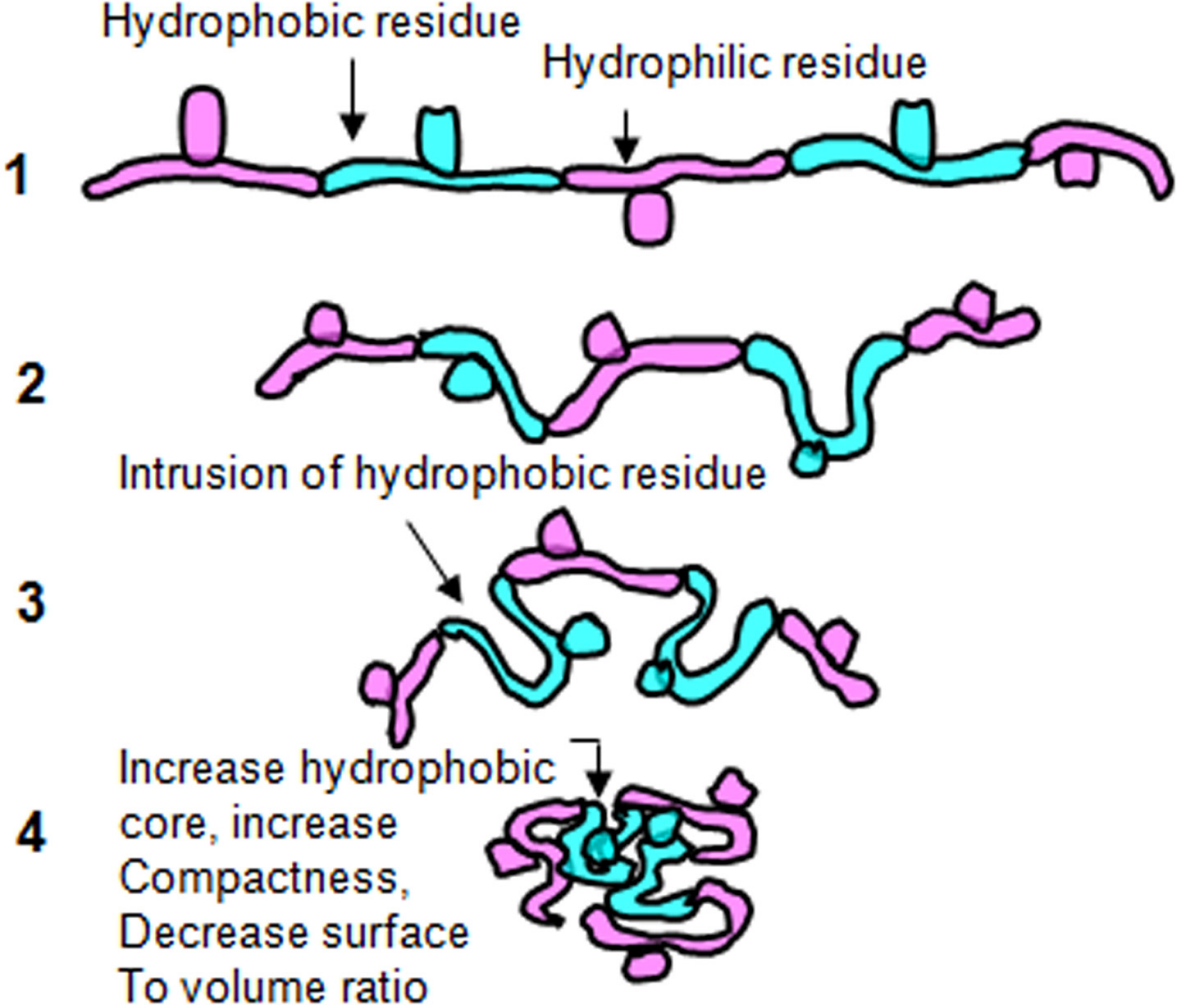
This figure demonstrates the possible fates of a polypeptide having a significant hydrophilic and hydrophobic residues combination. Greater hydrophobicity results in the intrusion of that part into the core of the molecule which generates a compact structure. That minimizes the surface area to volume ration and hence the possibility of water contact.

In Fig 8, a model diagram is shown to explain the formation of compact hydrophobic core in the thermophilic proteins. Several nonpolar hydrophobic portions of the peptide chains are intruded inward, leaving the surface of polar part and thus try to make compact core areas. This makes the proteins more globular in nature.

Present results suggest that the isoelectric points of mesophilic and thermophilic proteins are positively correlated (r = 0.93 and 0.84, respectively; p<0.001) to their corresponding charges. And their hydrophilicity is negatively associated with their corresponding hydrophobicity (r = -0.493, p<0.001 and r = -0.324, p<0.05) (Table 2). The salt bridge number of thermophilic proteins is found to be negatively correlated to its charge and total residue number (p<0.05). Thermophilic isoelectric point and charge were noticed to be positively associated with mesophilic isoelectric point and charge (p<0.05 –p<0.01) (Table 3).

**Table 2:** The statistical analyses were done by using the SPSS for Windows statistical software package (SPSS Inc., Chicago, IL, USA, 2001). Pearson correlation was utilized to evaluate the level of association of different physicochemical factors of 50 thermophilic and their homologue mesophilic proteins P value <0.05 is considered to be statistically significant.

Correlation	M-IPt vs M-C	M-Hpl vs M-Hpb	M-SB vs M-Hpl	T-IPt vs T-C	T-Hpl vs T-Hpb	T-SB vs T-IPt	T-SB vs T-C
**r**	0.930	-0.493	0.268	0.844	-0.324	-0.262	-0.326
**p**	0.001	0.001	0.059	0.001	0.022	0.066	0.021

M stands for mesophilic, T- thermophilic, IPt- isoelectric point, C- charge, Hpl- hydrophilic, Hpb- hydrophobic, SB- salt bridge.

**Table 3:** The statistical analyses were done by using the SPSS for Windows statistical software package (SPSS Inc., Chicago, IL, USA, 2001). Pearson correlation was utilized to evaluate the level of association of different physicochemical factors of 50 thermophilic and their homologue mesophilic proteins P value <0.05 is considered to be statistically significant.

Correlation	T-SB vs T-R	T-IPt vs M-IPt	T-IPt vs M-C	T-C vs M-IPt	T-C vs M-C	T-SB vs M-Hpb	T-R Vs M-R
**r**	-0.283	0.319	0.381	0.299	0.366	0.356	0.581
**p**	0.046	0.024	0.006	0.035	0.009	0.011	0.001

M stands for mesophilic, T- thermophilic, IPt- isoelectric point, C- charge, Hpl- hydrophilic, Hpb- hydrophobic, R- residue no.

## Discussion and Conclusion

A significant difference in the composition of the amino acids and their preferences between thermophilic (200) and mesophilic (200) proteins demonstrates the primary basis of the protein thermostability. In the present study, thermophilic proteins showed a consequential higher abundance of nonpolar amino acids of smaller volume, i.e. Ala, Gly and Val in their peptide chain which support some earlier evidence [18]. The appearance of the bulky volume aromatic residues is scanty in a larger number of thermophilic proteins in the present study. The role of these amino acids in the protein modifications is important for their structural and functional regulations. The aromatic residues such as tryptophan and tyrosine and their modifications have been shown to form some hot-spot region that lies at the protein-protein interface [32,33]. The histidine has an induced aromaticity and it is found to be distributed poorly/moderately in the most of the proteins of either type in our study. This amino acid participates in the catalytic activity of several enzymes. Forming a ‘catalytic triad’, the basic nitrogen of His helps in abstracting proton from several amino acids that initiates a nucleophilic attack [34,35]. During the catalytic processes the aromatic imidazole ring of His interacts with several metal cations (i.e. Zn^2+^, Ca^2+^), protonated amino acids (Lys^+^ and Arg^+^) or His^+^ can interact with aromatic amino acids (Phy, Tyr and Trp) or else utilizing μ-motifs, μ-μ stacking interactions (both aromatic rings remain face to face in μ plane) or few other interactions [36]. The His can also form hydrogen-μ, coordinate or hydrogen bond interactions. The coordinate bond and cation-μ interactions show the strongest interactive values [36]. In the protein structure, the transformation between the neutral His and His^+^ makes this amino acid versatile in character [36].

The abundance of the smaller volume residues and Pro may result in minimizing hindrance and entropy expenditure due to the side chain burial and favors looping and bending in proteins (Fig 1). This fact results in the formation of more compact core-region in the thermophilic protein structure [18,36,37]. In the thermophiles, the intrusions of the hydrophobic region in the different or a same plane of the peptide chain leave the polar region on the surface of the protein molecule [38,39]. This favors to form a more tangible globular structure of the protein molecule with versatilities in its function [39]. The withstanding ability of some proteins against heat stress is a naturally selected phenomenon [40]. And, the de-selection of the destabilizing polar amino acids in thermophilic proteins, as found in the present study has been a natural deliberation. It came into play under a significant evolutionary pressure to decrease the entropy generated due to the burial of polar side chains which results in a hierarchical flow of adaptation [18,41]. It is assumed that when a repertoire of mechanistic steps has been adopted for the protein thermostability, a more noncanonical pathway might have been implicated for the adaptation in the mesophilic proteins. The functionality of the protein is related more to its metabolic environment and to several intrinsic/ extrinsic factors. Sequence based studies on individual thermophile/mesophile ortholog pair suggests that only structure-based indices are poor determinant [42]. Further, this may indicate that the functional objectivity is more dependable for protein adaptability. In the present study, at certain % level, Met, Ile and Pro are comparatively higher occurring in the thermophilic proteins than that of their mesophilic counterparts suggesting the possible generation of hydrophobicity [43,44].

The polar residues are similar or slightly higher occurring in more mesophilic proteins (Fig 1). Polar amino acid with functional group-OH i.e. Ser, Thr and Tyr are moderately (6–8%) present in both the thermophilic and mesophilic proteins. It is noticed that when a lower percentage (0–4%) of Tyr is present in a large number of mesophilic proteins, a higher percentage (4–8%) of Tyr is occurring in a larger number of thermophiles [32,45,46]. Furthermore, keeping relevance to this data, our other result (Fig 6) reveals that thermophiles undergo a 25% higher abundance of Tyr phosphorylation then the corresponding mesophiles (p<0.05). This may indicate that a greater number of metabolic regulations by the possible phospho-modification of this residue might be occurring in thermophiles and higher order of animals [46,47]. It might offer a better paradigm of modifications towards adaptation process.

Being structurally similar to Cys, when Ser is occurring up to 10% in 95% of both types of proteins, Cys is occurring only up to 0–4% in more than 90% of both types of proteins. The finding in this control background states their relative differences in the functional properties and their mode to be utilized in the protein for specific adaptive purposes. This is reported that Cys can be modified and remain as-SH or-S-S- form for the enactment of the redox-switching of some of the protein molecules depending on the intracellular redox status. This status further can be regulated by the exogenous environment [48,49]. So, to enable Cys to perform in a sensitive and dependable manner in protein regulation, its abrupt abundance in proteins might have been evolutionary restricted. A slight higher abundance of Cys in some thermophiles (*χ* ^2^ = 19.99, p<0.01) may directly correlate to the organism’s complexity [50].

A greater percentage of occurrence of α-helix, β-sheet and left-handed helix in thermophilic proteins suggests their secondary structure to be configured in a better adaptive manner. It fulfills the functional conformity for withstanding the temperature-induced molecular instability [45]. Secondary structure analysis revealed that charged and aromatic amino acids were significant in sheet region of thermophiles [51]. More specifically, thermophilic β-galactosidases have a higher percentage of *α*-helix responsible for temperature tolerance [51] which is in line with the agreement of our present study of a large number of proteins. The propensities of the β-sheet, but not α-helix are demonstrated to differ between exposed and buried residues of the thermophilic proteins [52]. Further studies are required for a concluding remark from a large number of proteins at a global scale. The residues tyrosine and glycine, which are higher occurring in thermophiles in our study, and glutamine, show a significant increase in residency in alpha-helices of thermostable proteins [45].

Deviations of peptide bond from the planarity as noticed in the thermophiles in our study are suggested to make some precise functional conformity in the protein molecule [53]. The pragmatic analysis of protein conformation as a function of Φ, Ψ backbone dihedral angles show some deviations among those nonplanar structures. The *trans* peptide-form show larger deviation from the planarity [53,54]. The peptide-bond nonplanarity, are suggested to be less abundant in the active sites, but critically involved in the formation of tertiary structure in the protein molecule [53]. Two prominent resonance features contribute to the N-C double bond character and the planar peptide structure [55]. But the basis of the nonplanarity primarily arises in cyclic peptides and even in the linear peptide due to the presence of the bulky side-chains [56]. In relation to the adaptive evolutionary modifications, the provision for an introduction of peptide nonplanarity creates an opportunity for a protein not to succumb to some critical stress. Apart from the adjustment in the formal or conventional secondary structure, the generation of the nonplanarity in one or more peptide planar-sheet may help in further protein-modifications without interference to its active sites [53]. Beyond the extent of the compulsion of the peptide bond to remain in planarity, it may acquire a nonplanar structure depending on the nature of stress. In other words, it is indicative that the planarity may apparently serve as the natural savings of angles which helps at the time of crisis. Our points have been strongly justified by Karplus 1996 and Berkholz *et al*. 2011 with their comments that some of the protein, which becomes ‘frustrated’ due to some ‘hidden strain’, may be adaptively modified by an introduction of nonplanarity [57,53]. The deviation from the planar low-energy conformation [2,4] in proteins at the cost of thermodynamic energy may become more beneficial on the issues of withstanding an intrinsic strain (‘hidden strain’) or an extrinsic strain like higher temperature. This flexibility of rotation increases significantly with less energy cost in the proteins experiencing a very high temperature or in gas phase [58]. In addition to the nonplanarity, the occurrence of the small volume amino acids is advantageous in the thermostable protein folding. In line with our present study of higher Ala and Gly and moderate occurrence of Val in more thermophilic proteins, this work also indicated the role of these residues within the acceptable limits as inferred in the Ramachandran map [58].

In the protein, the *ω* torsion characterizes the peptide planarity, with *ω* = 180° as *trans* and *ω* = 0° as *cis* planar form [53]. In the present study, a 53% higher occurrence of *cis*-peptide bonds are evaluated in the mesophilic proteins. Reports reveal that a significant number of *cis* is noticed in the imide bond (pre-Pro) than the amide bonds [59] which further increases with the increasing resolutions during the protein structure determination [59]. The *trans* isomer (amide H bond) generates less steric repulsive barrier to the preceding C_α_ atom, then that of the *cis* isomer [60]. So, the protein backbone constitutes >99% of *trans* isomer. The higher number of *cis* is favored by the non planar peptide bonds in both thermophilic and mesophilic proteins which are noticed in the present study [61]. Both the *cis* and *trans* isomer of pre-Pro peptide bond are sterically hindered by the neighboring substitution [60]. The *cis* peptide bond is primarily found in the bends and turns which is noticed in our present results of higher loop and turns in mesophiles vs. thermophiles (45.46% vs. 32.38%, respectively). In case of *cis* imide bonds (pre-Pro) this correlation is so intense that it suggests some specific role of this bond in such protein structure [62]. The *cis* peptide bond is of both evolutionary and practical importance (physiological, pathological and enzyme catalytic processes). These bonds are also involved in intra and inter protein interactions by maintaining conformational dynamics with the help of proper looping and bending [63]. This finding justifies the higher abundance of *cis*-peptide in the different proteins in the present study [61].

The outliers are suggested to be contributed partially by glycine, proline and pre-proline, which minimizes the side chain burial and favors a better looping/bending and globular structure in the protein molecule [63,64]. The Ramachandran-plot for proline and glycine are different from the generic Ramachandran plot due to the presence of the pyrrolidine ring in proline and the absence of a C_β_ atom in glycine. These situations as possibly experienced in the current study, influence the flexibility and steric variations in the polypeptide backbone [63–65]. In our study, when the % of residues in the disallowed region does not vary significantly in thermophilic and mesophilic proteins (2.3% vs. 2.1%, respectively), the mean % of outlier showed a higher (12%) value in mesophilic proteins than their thermophilic homolog (4.45% vs. 3.97%). It may indicate a higher abundance of Pro and pre-Pro or more error in the concerned proteins. All these data are in agreement with the present findings of more loop/turn, outlier and *cis*-peptide in the mesophilic proteins. In addition, the finding of more consistent variation of outlier in the thermophilic proteins (range 2.3–4.9%) mean 3.97 vs. their homolog mesophiles (range 1.2–8.3%) mean 4.45 suggests the less inter-protein variability in the thermophilic proteins. This might have been possible due to more stratified and directed adaptive protein-modifications in thermophiles under a consistent selection pressure (increase in temperature) that generate more allelic fitness. An extensive genome-wide study of related species of Archaea and Bacteria suggests that, natural selection dominates to eliminate non-synonymous and synonymous mutation in thermophiles at a higher rate than in nonthermophiles [19]. This explains that the protein modification attributed by the mutational replacement of amino acids in thermophiles is very lower [19]. This work mainly dealt at the nucleotide level and our study deals with protein structural analysis still, it supports our hypothesis on the direct and active evolution by ‘strong purifying’ selection pressure generated due to the thermal stress. In contrast, the greater inter-protein variability in mesophiles is supported by the more varied occurrence of loops and turns in these proteins [66,67]. Unlike thermophilic proteins (where heat is employed as a defined confounding factors), the association of several factors is more stochastic in nature in the mesophilic proteins which have been modified in response to their individual and diverse adaptive/functional requirement [66–68].

A non-redundancy in the folding strategies in thermophilic and mesophilic proteins is observed in the present study. The greater usage of non-polar small amino acid and generation of hydrophobicity in thermophilic proteins has some advantageous thermodynamic concerns. The report reveals that the gain in enthalpy and the loss of entropy upon folding is lower in thermophiles suggesting its spontaneity in the modifications. This implies that the entropic stabilization is responsible for the heat adaptability in the thermophiles [17]. On the other hand, occupancy of the areas (initially covered with the water) by the non-polar side chains disrupts the highly dynamic hydrogen bonds between liquid water crystals [69,70]. But, the burial of those non-polar side chains and further their aggregation towards the core region reduce the exposed surface-area to water which minimize these disruptive effects (Fig 5). These disruptive effects are further compensated by the greater occurrence of the salt bridges in the thermophiles which is noticed in the present study [26]. About 60% of the thermophiles show a higher abundance of the salt bridges. The report suggests that salt bridges and main chain hydrogen bonds are increased in the majority of the thermophilic proteins [26]. In this regard, Gly has some specific role in the thermodynamic equilibrium of the thermophiles. As for example, in case of thermophilic form of RNase H, Gly insertion plays a major role in modulating conformational dynamics of this protein structure [71]. It may be true for other proteins also. Due to the lack of a C_β_ atom, Gly occupies a major space of the Ramachandran plot compared to other amino acids. This structural plasticity appears to alleviate the unfavorable interactions in the transition state in RNase H suggesting the more responsible role of Gly in thermostability [71].

A slight acidic isoelectric point (pI) in thermophilic proteins and its net negative charge (-0.6, at neutral pH) (Fig 3) suggest that these proteins will carry a more acidic group (Asp and Glu) (Fig 1). The pI of a number of experimental proteins has been shown with bimodal values and in a slight acidic range [72]. The thermo-stabilizing role of Asp and Glu, and the electrostatic interactions have been revealed in the thermophilic ribosomal protein L30e from *Thermococcus celer* [73]. In contrary, in case of mesophilic proteins, average pI is slightly basic, and the average net charge being positive (1), the protein will carry more basic side chain (i.e. Arg, *χ* ^2^ = 41.56, p<0.001) (Fig 1). The experimental data on the stability of a well studied protein ribonuclease Sa reveals that due to the excess acidic residues in its chain (7 Asp, 5 Glu vs. 2 His, 0 Lys, 5 Arg), it shows the pI at 3.5 and net charge ~ -7 (at pH 7), but one or more replacement of Asp and/or Glu with Lys increasingly reverses these parameters up to, pI >10 and charge = +3 after a total 5 replacements [74]. This strongly supports our result from a total 100 proteins. The pI, a determinant of protein solubility and stability is of the great practical importance in some disease condition, i.e. Alzheimer’s [75], in the development of a recombinant therapeutic proteins such as fast acting Lys-Pro insulin [76], in X-ray crystallographic and other studies. The report reveals that a greater number of Glu is preferentially located and form ion-pairs on the surface of the thermophilic proteins [77]. This data is consistent with our result of thermostability conferred by more GLU-LYS and GLU-HIS salt bridges in thermophilic proteins. Kawamura *et al*. (1997) described that the disruption of the GLU-LYS salt bridge in a DNA binding protein, HU from *Bacillus stearothermophilus* significantly reduced its thermal stability [78]. The GLU-ARG dyad is noticed to be higher in the mesophilic protein in our study. The Arg is reported to form ion pairs in protein network [77]. Nevertheless, the most of these previous studies were conducted with a single purified protein of interests. The study of protein stability is of great relevance to its biotechnological application. An important strategy to augment protein stability is to optimize the charge-charge interaction in it. Correlation data also support the result that the isoelectric points of proteins of both groups are positively correlated (r = 0.93 and 0.84, respectively, p<0.001) to their corresponding charges. And the significant negative association between protein hydrophobicity and hydrophilicity suggest that either property was evolved by the expense of the other.

The thermophilic proteins are noticed to be little smaller in length/ volume (mean 301 vs. 328 residues in 50 thermophiles and 50 mosophiles, respectively; Table 1 and Fig 5), notwithstanding found competent against heat stress. These findings are suggested to have more evolutionary concern than only physical thermo-stabilization in protein molecules [79]. The lost length of the polypeptide chain might or might not enrich the protein with some extra hydrophobic pockets, but at the same time, it would unequivocally increase some degrees of stringency due to the introduction of new peptide-bond planarity. To compensate the shortcomings on the length issues, introduction with certain extent of nonplanarity as we noticed might help the protein for better packaging of existing hydrophobic core for more intense avoidance of water, hence heat exposure. In addition, increasing compactness or closely ness induces extra molecular-affairs, i.e. charge-charge interactions and salt bridges, and helps the proteins to transform in a vigorous ensemble of several other weak interactions to acquire further stabilization. Hydrophobicity, compactness and other intrinsic factors support the entropic stabilization in the thermophilic proteins. This situation results in their increase in ΔG and melting temperature [80]. The increased hydrophobicity and decreased surface area to volume ratio (lesser contact with water) enable thermophilic proteins to experience lower magnitude of heat associated destabilizing forces. In conclusion, structural diversity offers an increased number of interaction ability, and opening of several newer avenues for more metabolic opportunities. Increased new possibilities of protein interaction, in turn, support the evolutionary processes. It is one of the bases of the thermophilic adaptations in proteins.

## Supporting Information

S1 File Table A represents the two hundred thermophilic and two hundred mesophilic proteins, Table B represents the fifty thermophilic proteins and Table C represents the fifty mesophilic proteins. (DOC)
