# Supplementary material for: Protein Thermostability Is Owing to Their Preferences to Non-Polar Smaller Volume Amino Acids, Variations in Residual Physico-Chemical Properties and More Salt-Bridges
Source: PLoS One. 2015 Jul 15;10(7):e0131495. doi: 10.1371/journal.pone.0131495 (PMC4503463; doi:10.1371/journal.pone.0131495)
Supplement: S1 File — (DOC) [file pone.0131495.s001.doc]

*PLOS ONE* Supporting Information (S1 File)

**Title: Protein Thermostability Is Owing to Their Preferences to Non-Polar Smaller Volume Amino Acids, Variations in Residual Physico-Chemical Properties and More Salt-Bridges**

Table A. Two hundred thermophilic and Two hundred mesophilic proteins

| Name of protein | 1-deoxy-D-xylulose-5-phosphate synthase |
| --- | --- |
| Code(meso) | P77488 |
| Code(thermo) | Q75TB7 |

| Name of protein | 3-dehydroquinate dehydratase |
| --- | --- |
| Code(meso) | P05194 |
| Code(thermo) | Q9YEK1 |

| Name of protein | 3-dehydroquinate synthase |
| --- | --- |
| Code(meso) | P07639 |
| Code(thermo) | Q5KXU5 |

| Name of protein | 3-phosphoshikimate 1-carboxyvinyltransferase |
| --- | --- |
| Code(meso) | P0A6D3 |
| Code(thermo) | Q5KXV5 |

| Name of protein | 60 kDa heat shock protein |
| --- | --- |
| Code(meso) | Q5NVM5 |
| Code(thermo) | Q5L3E6 |

| Name of protein | Adenosylmethionine-8-amino-7-oxononanoateaminotransferase |
| --- | --- |
| Code(meso) | C6WDB9 |
| Code(thermo) | B7GHM5 |

| Name of protein | HUMAN Adenylosuccinate lyase |
| --- | --- |
| Code(meso) | P30566 |
| Code(thermo) | O58582 |

| Name of protein | HUMAN Adenylosuccinate synthetase isozyme |
| --- | --- |
| Code(meso) | P30520 |
| Code(thermo) | Q5KU76 |

| Name of protein | Arginine biosynthesis bifunctional protein ArgJ |
| --- | --- |
| Code(meso) | E6YS87 |
| Code(thermo) | Q5L1V4 |

| Name of protein | Arginine decarboxylase proenzyme |
| --- | --- |
| Code(meso) | A8AAB6 |
| Code(thermo) | Q9YG22 |

| Name of protein | Arginine represso |
| --- | --- |
| Code(meso) | P0A6D0 |
| Code(thermo) | A4XKP7 |

| Name of protein | Argininosuccinate lyase |
| --- | --- |
| Code(meso) | P04424 |
| Code(thermo) | Q5KW95 |

| Name of protein | Argininosuccinate lyase |
| --- | --- |
| Code(meso) | P22675 |
| Code(thermo) | B7GGR5 |

| Name of protein | ATP synthase subunit 5, mitochondrial |
| --- | --- |
| Code(meso) | P09457 |
| Code(thermo) | Q5KUJ2 |

| Name of protein | ATP synthase subunit 5, mitochondrial |
| --- | --- |
| Code(meso) | O74190 |
| Code(thermo) | Q5KUJ4 |

| Name of protein | ATP synthase subunit alpha |
| --- | --- |
| Code(meso) | P25705 |
| Code(thermo) | B7GMF5 |

| Name of protein | ATP synthase subunit b |
| --- | --- |
| Code(meso) | P24539 |
| Code(thermo) | Q5KUI9 |

| Name of protein | ATP synthase subunit c |
| --- | --- |
| Code(meso) | P68699 |
| Code(thermo) | B7GMF8 |

| Name of protein | ATP synthase subunit delta |
| --- | --- |
| Code(meso) | P0ABA4 |
| Code(thermo) | Q5KUJ0 |

| Name of protein | ATP synthase subunit delta |
| --- | --- |
| Code(meso) | A5HY49 |
| Code(thermo) | A4XKX3 |

| Name of protein | ATP synthase subunit epsilon |
| --- | --- |
| Code(meso) | P56381 |
| Code(thermo) | B7GMF2 |

| Name of protein | Chorismate synthase |
| --- | --- |
| Code(meso) | P28777 |
| Code(thermo) | Q5KXU4 |

| Name of protein | 3-dehydroquinate dehydratase |
| --- | --- |
| Code(meso) | Q8DUW4 |
| Code(thermo) | Q5KY94 |

| Name of protein | 3-dehydroquinate synthase |
| --- | --- |
| Code(meso) | Q9X5D2 |
| Code(thermo) | Q8DKS3 |

| Name of protein | HUMAN Phosphatidylserine decarboxylase proenzyme |
| --- | --- |
| Code(meso) | Q9UG56 |
| Code(thermo) | Q5KWX3 |

| Name of protein | Phosphoadenosine phosphosulfate reductase |
| --- | --- |
| Code(meso) | P17854 |
| Code(thermo) | B7GG21 |

| Name of protein | Phosphoadenosine phosphosulfate reductase |
| --- | --- |
| Code(meso) | Q8Z460 |
| Code(thermo) | Q5L2X9 |

| Name of protein | Phosphopentomutase |
| --- | --- |
| Code(meso) | P0A6K6 |
| Code(thermo) | Q5KXI7 |

| Name of protein | Phosphoribosylformylglycinamidine |
| --- | --- |
| Code(meso) | P08178 |
| Code(thermo) | Q5L3D0 |

| Name of protein | Phosphoribosylformylglycinamidine synthase |
| --- | --- |
| Code(meso) | Q8G180 |
| Code(thermo) | Q5L3D3 |

| Name of protein | Phosphoribosylformylglycinamidine synthase |
| --- | --- |
| Code(meso) | P0A5T8 |
| Code(thermo) | B7GFT8 |

| Name of protein | 3-phosphoshikimate 1-carboxyvinyltransferase |
| --- | --- |
| Code(meso) | P22487 |
| Code(thermo) | Q5KXV5 |

| Name of protein | HUMAN ATP synthase subunit epsilon |
| --- | --- |
| Code(meso) | P56381 |
| Code(thermo) | Q5KUV3 |

| Name of protein | PROBABLE BIFUNCTIONAL ENZYME CYSN/CYSC: SULFATE ADENYLTRANSFERASE (SUBUNIT 1) |
| --- | --- |
| Code(meso) | Q7U089 |
| Code(thermo) | Q9YCR6 |

| Name of protein | Probable deoxycytidine triphosphate deaminase |
| --- | --- |
| Code(meso) | Q2NFH1 |
| Code(thermo) | Q9YFA8 |

| Name of protein | Protein CrcB homolog |
| --- | --- |
| Code(meso) | P63861 |
| Code(thermo) | Q5KWE9 |

| Name of protein | Protein CrcB homolog 2 |
| --- | --- |
| Code(meso) | Q8ZDB2 |
| Code(thermo) | Q5KWE8 |

| Name of protein | Probable adenylyltransferase/sulfurtransferase MoeZ |
| --- | --- |
| Code(meso) | Q7D5X9 |
| Code(thermo) | Q9YBL2 |

| Name of protein | Protoheme IX farnesyltransferase |
| --- | --- |
| Code(meso) | P0AEA5 |
| Code(thermo) | Q5L114 |

| Name of protein | Pseudouridine-5'-phosphate glycosidase |
| --- | --- |
| Code(meso) | P33025 |
| Code(thermo) | Q5L078 |

| Name of protein | Purine nucleoside phosphorylase |
| --- | --- |
| Code(meso) | P0ABP8 |
| Code(thermo) | Q5KZM1 |

| Name of protein | Putative cobalt-precorrin-6A synthase [deacetylating] |
| --- | --- |
| Code(meso) | Q97JA9 |
| Code(thermo) | Q5KZ00 |

| Name of protein | Putative competence-damage inducible protei |
| --- | --- |
| Code(meso) | Q65JF3 |
| Code(thermo) | Q5L0F7 |

| Name of protein | Putative competence-damage inducible protein |
| --- | --- |
| Code(meso) | P46323 |
| Code(thermo) | B7GJN4 |

| Name of protein | Putative L-asparaginase I |
| --- | --- |
| Code(meso) | Q6LP01 |
| Code(thermo) | O57971 |

| Name of protein | RNA-binding protein AU-1 |
| --- | --- |
| Code(meso) | Q4J9H1 |
| Code(thermo) | Q9Y9F2 |

| Name of protein | Shikimate kinase |
| --- | --- |
| Code(meso) | P37944 |
| Code(thermo) | Q5KY95 |

| Name of protein | Shikimate kinase |
| --- | --- |
| Code(meso) | Q3ZZK9 |
| Code(thermo) | Q8DKH7 |

| Name of protein | Sulfite reductase [NADPH] hemoprotein beta-component |
| --- | --- |
| Code(meso) | P17846 |
| Code(thermo) | Q5L041 |

| Name of protein | Sulfite reductase [NADPH] hemoprotein beta-component |
| --- | --- |
| Code(meso) | Q8X7U2 |
| Code(thermo) | B7GJU8 |

| Name of protein | Pantothenate kinase |
| --- | --- |
| Code(meso) | Q9H999 |
| Code(thermo) | Q5L3T0 |

| Name of protein | Uncharacterized oxidoreductase |
| --- | --- |
| Code(meso) | C2ZHL9 |
| Code(thermo) | Q5L2G3 |

| Name of protein | HUMAN Uroporphyrinogen decarboxylase |
| --- | --- |
| Code(meso) | P06132 |
| Code(thermo) | Q5L284 |

| Name of protein | 2,3-bisphosphoglycerate-independent phosphoglycerate mutase |
| --- | --- |
| Code(meso) | Q81X77 |
| Code(thermo) | Q9YBI2 |

| Name of protein | 2,3-bisphosphoglycerate-independent phosphoglycerate mutase2 |
| --- | --- |
| Code(meso) | P30792 |
| Code(thermo) | O57742 |

| Name of protein | RAT Coagulation factorX |
| --- | --- |
| Code(meso) | Q63207 |
| Code(thermo) | D3DJ41 |

| Name of protein | L-asparaginase, putative |
| --- | --- |
| Code(meso) | C9R6E4 |
| Code(thermo) | O57971 |

| Name of protein | 3-dehydroquinate dehydratase |
| --- | --- |
| Code(meso) | P24670 |
| Code(thermo) | Q8DLJ7 |

| Name of protein | Arginine biosynthesis bifunctional protein |
| --- | --- |
| Code(meso) | P74122 |
| Code(thermo) | Q5L1V4 |

| Name of protein | Arginine biosynthesis bifunctional protein |
| --- | --- |
| Code(meso) | P36843 |
| Code(thermo) | Q07908 |

| Name of protein | Amino-acid acetyltransferase |
| --- | --- |
| Code(meso) | B9W7S3 |
| Code(thermo) | P96137 |

| Name of protein | HUMAN Argininosuccinate lyase |
| --- | --- |
| Code(meso) | P04424 |
| Code(thermo) | B7GGR5 |

| Name of protein | Arginine repressor |
| --- | --- |
| Code(meso) | Q9Z8Z1 |
| Code(thermo) | Q5KXB1 |

| Name of protein | Arginine biosynthesis bifunctional protein |
| --- | --- |
| Code(meso) | Q92MJ1 |
| Code(thermo) | Q9Z4S1 |

| Name of protein | Arginine biosynthesis bifunctional protein |
| --- | --- |
| Code(meso) | Q8DHN4 |
| Code(thermo) | P62057 |

| Name of protein | Acetylglutamate/acetylaminoadipate kinase |
| --- | --- |
| Code(meso) | A4WJI0 |
| Code(thermo) | O59398 |

| Name of protein | 23S rRNA (uracil(747)-C(5))-methyltransferase |
| --- | --- |
| Code(meso) | Q9CKK7 |
| Code(thermo) | O58994 |

| Name of protein | N-acetyl-gamma-glutamyl-phosphate reductase |
| --- | --- |
| Code(meso) | Q8ZA86 |
| Code(thermo) | P59312 |

| Name of protein | 3-dehydroquinate dehydratase |
| --- | --- |
| Code(meso) | Q829X9 |
| Code(thermo) | A4XLN1 |

| Name of protein | 3-dehydroquinate dehydratase |
| --- | --- |
| Code(meso) | B6JHG1 |
| Code(thermo) | B7GHE3 |

| Name of protein | N-acetyl-gamma-glutamyl-phosphate/N-acetyl-gamma-aminoadipyl-phosphate reductase |
| --- | --- |
| Code(meso) | Q9RVQ9 |
| Code(thermo) | O59397 |

| Name of protein | Shikimate kinase |
| --- | --- |
| Code(meso) | B0BSJ7 |
| Code(thermo) | A4XLN3 |

| Name of protein | Arginine decarboxylase proenzyme |
| --- | --- |
| Code(meso) | C3N6F7 |
| Code(thermo) | Q9UWU1 |

| Name of protein | Shikimate kinase |
| --- | --- |
| Code(meso) | P56073 |
| Code(thermo) | Q9YEK6 |

| Name of protein | Shikimate dehydrogenase |
| --- | --- |
| Code(meso) | Q58484 |
| Code(thermo) | Q8DLA6 |

| Name of protein | shikimate dehydrogenase |
| --- | --- |
| Code(meso) | P46240 |
| Code(thermo) | A4XID4 |

| Name of protein | Arginine repressor |
| --- | --- |
| Code(meso) | P0A1B3 |
| Code(thermo) | A4XKP7 |

| Name of protein | N-acetyl-gamma-glutamyl-phosphate reductase |
| --- | --- |
| Code(meso) | Q8X732 |
| Code(thermo) | Q5L1V5 |

| Name of protein | N-acetyl-gamma-glutamyl-phosphate reductase |
| --- | --- |
| Code(meso) | A3M5Z6 |
| Code(thermo) | A4XJN9 |

| Name of protein | 3-dehydroquinate synthase |
| --- | --- |
| Code(meso) | Q18DY6 |
| Code(thermo) | Q9YEJ9 |

| Name of protein | Argininosuccinate lyase |
| --- | --- |
| Code(meso) | Q46104 |
| Code(thermo) | Q8DLW0 |

| Name of protein | Argininosuccinate lyase |
| --- | --- |
| Code(meso) | B7GTN9 |
| Code(thermo) | Q9UX32 |

| Name of protein | Argininosuccinate synthase |
| --- | --- |
| Code(meso) | P0A6E4 |
| Code(thermo) | B7GGR4 |

| Name of protein | Argininosuccinate synthase |
| --- | --- |
| Code(meso) | P00966 |
| Code(thermo) | A4XKG4 |

| Name of protein | N-acetyl-gamma-glutamyl-phosphate reductase |
| --- | --- |
| Code(meso) | P11446 |
| Code(thermo) | Q9Z4S2 |

| Name of protein | Argininosuccinate synthase |
| --- | --- |
| Code(meso) | Q5KW94 |
| Code(thermo) | Q5KW94 |

| Name of protein | Argininosuccinate synthase |
| --- | --- |
| Code(meso) | Q9X2A1 |
| Code(thermo) | Q9UX31 |

| Name of protein | Argininosuccinate synthase |
| --- | --- |
| Code(meso) | A5I5A4 |
| Code(thermo) | Q8DKY7 |

| Name of protein | 7-cyano-7-deazaguanine tRNA-ribosyltransferase |
| --- | --- |
| Code(meso) | B1L6M8 |
| Code(thermo) | Q9YAC2 |

| Name of protein | 7-cyano-7-deazaguanine tRNA-ribosyltransferase |
| --- | --- |
| Code(meso) | B6YUR8 |
| Code(thermo) | Q57878 |

| Name of protein | Arginine decarboxylase proenzyme |
| --- | --- |
| Code(meso) | C3MWN7 |
| Code(thermo) | Q9YG22 |

| Name of protein | Acetylornithine aminotransferase |
| --- | --- |
| Code(meso) | Q9PIR7 |
| Code(thermo) | P59322 |

| Name of protein | N-acetyl-gamma-glutamyl-phosphate/N-acetyl-gamma-aminoadipyl-phosphate reductase |
| --- | --- |
| Code(meso) | A9A1K6 |
| Code(thermo) | O50146 |

| Name of protein | N-acetyl-gamma-glutamyl-phosphate reductase, chloroplastic |
| --- | --- |
| Code(meso) | Q6AV34 |
| Code(thermo) | P96136 |

| Name of protein | Acetylglutamate kinase |
| --- | --- |
| Code(meso) | P68729 |
| Code(thermo) | P59303 |

| Name of protein | Acetylornithine/acetyl-lysine aminotransferase |
| --- | --- |
| Code(meso) | Q7SI94 |
| Code(thermo) | Q9YBY6 |

| Name of protein | N-acetyl-gamma-glutamyl-phosphate/N-acetyl-gamma-aminoadipyl-phosphate reductase |
| --- | --- |
| Code(meso) | C3MZY9 |
| Code(thermo) | Q9YBY8 |

| Name of protein | Acetaldehyde dehydrogenase |
| --- | --- |
| Code(meso) | Q52060 |
| Code(thermo) | Q764S1 |

| Name of protein | Acetate kinase OS=Escherichia coli |
| --- | --- |
| Code(meso) | P0A6A3 |
| Code(thermo) | B7GGR2 |

| Name of protein | Acetate kinase |
| --- | --- |
| Code(meso) | Q8ZDJ6 |
| Code(thermo) | A4XL40 |

| Name of protein | HUMAN cAMP-dependent protein kinase catalytic subunit |
| --- | --- |
| Code(meso) | P51817 |
| Code(thermo) | Q5KW66 |

| Name of protein | Acetate kinase |
| --- | --- |
| Code(meso) | P57866 |
| Code(thermo) | Q8DGH7 |

| Name of protein | Acetyl-coenzyme A carboxylase carboxyl transferase subunit beta |
| --- | --- |
| Code(meso) | P0A9Q5 |
| Code(thermo) | B7GGT0 |

| Name of protein | Acetyl-coenzyme A carboxylase carboxyl transferase subunit alpha |
| --- | --- |
| Code(meso) | P0ABD5 |
| Code(thermo) | Q5KWB0 |

| Name of protein | Acetyl-coenzyme A carboxylase carboxyl transferase subunit beta, chloroplastic |
| --- | --- |
| Code(meso) | P56765 |
| Code(thermo) | Q8DJB6 |

| Name of protein | Acetyl-coenzyme A carboxylase carboxyl transferase subunit beta |
| --- | --- |
| Code(meso) | P18823 |
| Code(thermo) | B7GGS9 |

| Name of protein | Acetyl-coenzyme A carboxylase carboxyl transferase subunit beta |
| --- | --- |
| Code(meso) | Q9HZA7 |
| Code(thermo) | Q5KWA9 |

| Name of protein | Acetyl-coenzyme A synthetase |
| --- | --- |
| Code(meso) | P27550 |
| Code(thermo) | Q8DKH2 |

| Name of protein | Acetylglutamate kinase |
| --- | --- |
| Code(meso) | Q9HTN2 |
| Code(thermo) | A4XJN8 |

| Name of protein | Amino-acid acetyltransferase |
| --- | --- |
| Code(meso) | P0A6C5 |
| Code(thermo) | Q5L1V3 |

| Name of protein | Acetylglutamate/acetylaminoadipate kinase |
| --- | --- |
| Code(meso) | C3NF41 |
| Code(thermo) | Q9YBY9 |

| Name of protein | Actin-related protein 3 |
| --- | --- |
| Code(meso) | P47117 |
| Code(thermo) | P10365 |

| Name of protein | Acyl carrier protein |
| --- | --- |
| Code(meso) | D3QU85 |
| Code(thermo) | Q5L0Q4 |

| Name of protein | Acyl-CoA-binding domain-containing protein 2 |
| --- | --- |
| Code(meso) | Q9STP8 |
| Code(thermo) | Q8DHS3 |

| Name of protein | Acylamino-acid-releasing enzyme |
| --- | --- |
| Code(meso) | Q1RH50 |
| Code(thermo) | Q9YBQ2 |

| Name of protein | Acylphosphatase-1 |
| --- | --- |
| Code(meso) | P07311 |
| Code(thermo) | Q9YBK7 |

| Name of protein | Acylphosphatase-2 |
| --- | --- |
| Code(meso) | P00818 |
| Code(thermo) | Q5L2Y4 |

| Name of protein | HUMAN Acylphosphatase-2 |
| --- | --- |
| Code(meso) | P14621 |
| Code(thermo) | P84142 |

| Name of protein | Adenine deaminase |
| --- | --- |
| Code(meso) | P39761 |
| Code(thermo) | Q5KY53 |

| Name of protein | Adenine phosphoribosyltransferase |
| --- | --- |
| Code(meso) | P69503 |
| Code(thermo) | A4XI79 |

| Name of protein | MOUSE Adenine phosphoribosyltransferase |
| --- | --- |
| Code(meso) | P08030 |
| Code(thermo) | Q5KWS2 |

| Name of protein | Adenine phosphoribosyltransferase |
| --- | --- |
| Code(meso) | A3MYY8 |
| Code(thermo) | Q8DGH9 |

| Name of protein | ECOLI Alanine racemase, biosynthetic |
| --- | --- |
| Code(meso) | P0A6B4 |
| Code(thermo) | B7GFP5 |

| Name of protein | Alanine racemase |
| --- | --- |
| Code(meso) | Q6LX41 |
| Code(thermo) | Q9F8I0 |

| Name of protein | Alanine racemase |
| --- | --- |
| Code(meso) | Q8RSU9 |
| Code(thermo) | Q5L3G4 |

| Name of protein | _MOUSE Alanyl-tRNA editing protein Aarsd1 |
| --- | --- |
| Code(meso) | Q3THG9 |
| Code(thermo) | Q8Q0A4 |

| Name of protein | RAT Alcohol dehydrogenase 4 |
| --- | --- |
| Code(meso) | Q64563 |
| Code(thermo) | P42328 |

| Name of protein | Aliphatic amidase regulator |
| --- | --- |
| Code(meso) | P10932 |
| Code(thermo) | Q9L543 |

| Name of protein | Aliphatic amidase regulator2 |
| --- | --- |
| Code(meso) | P10933 |
| Code(thermo) | Q9L544 |

| Name of protein | Aliphatic amidase regulator x |
| --- | --- |
| Code(meso) | P10931 |
| Code(thermo) | Q9L545 |

| Name of protein | Alpha-amylase inhibitor |
| --- | --- |
| Code(meso) | P83048 |
| Code(thermo) | O57932 |

| Name of protein | Alpha-amylase inhibitor AAI |
| --- | --- |
| Code(meso) | P80403 |
| Code(thermo) | P86331 |

| Name of protein | Alpha-amylase/trypsin inhibitor |
| --- | --- |
| Code(meso) | P16969 |
| Code(thermo) | P06279 |

| Name of protein | Aminopeptidase 2, mitochondrial |
| --- | --- |
| Code(meso) | P32454 |
| Code(thermo) | P23341 |

| Name of protein | Anhydro-N-acetylmuramic acid kinase |
| --- | --- |
| Code(meso) | P77570 |
| Code(thermo) | Q8DGC2 |

| Name of protein | Aqualysin-1 OS=Streptomyces |
| --- | --- |
| Code(meso) | **B4V9G8** |
| Code(thermo) | P08594 |

| Name of protein | Archaemetzincin |
| --- | --- |
| Code(meso) | C3MVY2 |
| Code(thermo) | O59484 |

| Name of protein | Diaminobutyrate--2-oxoglutarate aminotransferase |
| --- | --- |
| Code(meso) | Q9Z3R2 |
| Code(thermo) | O58489 |

| Name of protein | HUMAN Putative ATP-dependent Clp protease proteolytic subunit |
| --- | --- |
| Code(meso) | Q16740 |
| Code(thermo) | B7GL34 |

| Name of protein | Cardiolipin synthase |
| --- | --- |
| Code(meso) | P0A6H8 |
| Code(thermo) | Q5L1S5 |

| Name of protein | HUMAN CCA tRNA nucleotidyltransferase 1, mitochondria |
| --- | --- |
| Code(meso) | Q96Q11 |
| Code(thermo) | Q5KXX0 |

| Name of protein | Cell division protein DivIB |
| --- | --- |
| Code(meso) | Q894C1 |
| Code(thermo) | Q5L0X5 |

| Name of protein | Chaperone protein dnaK2 |
| --- | --- |
| Code(meso) | Q826F6 |
| Code(thermo) | Q8DI58 |

| Name of protein | 2-succinyl-6-hydroxy-2,4-cyclohexadiene-1-carboxylate synthase |
| --- | --- |
| Code(meso) | Q7CJ75 |
| Code(thermo) | Q9YEL4 |

| Name of protein | Cobalt transport protein CbiM |
| --- | --- |
| Code(meso) | D5AUZ9 |
| Code(thermo) | B7GLU2 |

| Name of protein | D-alanine--D-alanine ligase B |
| --- | --- |
| Code(meso) | P07862 |
| Code(thermo) | Q5L3H2 |

| Name of protein | DEAD-box ATP-dependent RNA helicase CshA |
| --- | --- |
| Code(meso) | P96614 |
| Code(thermo) | Q5L3G9 |

| Name of protein | Dephospho-CoA kinase |
| --- | --- |
| Code(meso) | Q9Z7U3 |
| Code(thermo) | Q5KWC4 |

| Name of protein | Diaminopimelate epimerase |
| --- | --- |
| Code(meso) | P0A6K1 |
| Code(thermo) | Q5KVP4 |

| Name of protein | ECOLI Dihydroorotase |
| --- | --- |
| Code(meso) | P05020 |
| Code(thermo) | B7GFA5 |

| Name of protein | HUMAN Diphthine synthase |
| --- | --- |
| Code(meso) | Q9H2P9 |
| Code(thermo) | Q9YDI2 |

| Name of protein | DNA-binding protein 7d |
| --- | --- |
| Code(meso) | P13123 |
| Code(thermo) | P61991 |

| Name of protein | D-tyrosyl-tRNA(Tyr) deacylase |
| --- | --- |
| Code(meso) | P0A6M4 |
| Code(thermo) | Q5KWS4 |

| Name of protein | Energy-coupling factor transporter ATP-binding protein EcfA 2 |
| --- | --- |
| Code(meso) | Q8NVB5 |
| Code(thermo) | Q5L3Q9 |

| Name of protein | FMN-dependent NADH-azoreductase |
| --- | --- |
| Code(meso) | P41407 |
| Code(thermo) | Q5KUC3 |

| Name of protein | ECOLI Fructose-bisphosphate aldolase class 2 |
| --- | --- |
| Code(meso) | P0AB71 |
| Code(thermo) | O57840 |

| Name of protein | AGRT5 Glucoamylase |
| --- | --- |
| Code(meso) | Q7CVZ6 |
| Code(thermo) | P29761 |

| Name of protein | Glutamate N-acetyltransferase 2 |
| --- | --- |
| Code(meso) | Q53940 |
| Code(thermo) | Q57645 |

| Name of protein | ECOLI GTPase Der |
| --- | --- |
| Code(meso) | P0A6P5 |
| Code(thermo) | Q5KXT0 |

| Name of protein | RICAH Heme A synthase |
| --- | --- |
| Code(meso) | A8GMQ9 |
| Code(thermo) | Q5L115 |

| Name of protein | Heme/copper-type cytochrome/quinol oxidase, subunit 2 |
| --- | --- |
| Code(meso) | F0QJS3 |
| Code(thermo) | Q9YDX7 |

| Name of protein | Heme/copper-type cytochrome/quinol oxidase, subunit 3 |
| --- | --- |
| Code(meso) | F7S0F4 |
| Code(thermo) | Q9YDX4 |

| Name of protein | Quinol oxidase subunit 1/3 |
| --- | --- |
| Code(meso) | P39481 |
| Code(thermo) | Q9YDX6 |

| Name of protein | Holo-[acyl-carrier-protein] synthase |
| --- | --- |
| Code(meso) | Q81JG3 |
| Code(thermo) | B7GIY1 |

| Name of protein | Holo-[acyl-carrier-protein] synthase |
| --- | --- |
| Code(meso) | P0A4W8 |
| Code(thermo) | A4XIB7 |

| Name of protein | Holo-[acyl-carrier-protein] synthase |
| --- | --- |
| Code(meso) | Q0SPF4 |
| Code(thermo) | Q5L3G7 |

| Name of protein | L-arabinose isomerase |
| --- | --- |
| Code(meso) | B3PD57 |
| Code(thermo) | B7GGW0 |

| Name of protein | L-arabinose isomerase OS=Yersinia pestis |
| --- | --- |
| Code(meso) | P58540 |
| Code(thermo) | Q5KYP7 |

| Name of protein | MOUSE Leucyl-cystinyl aminopeptidase |
| --- | --- |
| Code(meso) | Q8C129 |
| Code(thermo) | P95928 |

| Name of protein | Magnesium-protoporphyrin IX monomethyl ester [oxidative] cyclase |
| --- | --- |
| Code(meso) | Q5MZZ2 |
| Code(thermo) | Q8DJ05 |

| Name of protein | Anaerobic magnesium-protoporphyrin IX monomethyl ester [oxidative] cyclase |
| --- | --- |
| Code(meso) | P26168 |
| Code(thermo) | Q8DI68 |

| Name of protein | Malonyl-CoA O-methyltransferase BioC |
| --- | --- |
| Code(meso) | P12999 |
| Code(thermo) | B7GHW9 |

| Name of protein | HUMAN Methionine aminopeptidase 1D, mitochondrial |
| --- | --- |
| Code(meso) | Q6UB28 |
| Code(thermo) | O58362 |

| Name of protein | N-acetyldiaminopimelate deacetylase |
| --- | --- |
| Code(meso) | A0RHZ2 |
| Code(thermo) | Q5L145 |

| Name of protein | N-acetyl-gamma-glutamyl-phosphate reductase |
| --- | --- |
| Code(meso) | P11446 |
| Code(thermo) | Q5L1V5 |

| Name of protein | Probable ATP-dependent transporter |
| --- | --- |
| Code(meso) | P48255 |
| Code(thermo) | P35020 |

| Name of protein | NADP-dependent alcohol dehydrogenase 6 |
| --- | --- |
| Code(meso) | Q04894 |
| Code(thermo) | P14941 |

| Name of protein | N-glycosylase/DNA lyase |
| --- | --- |
| Code(meso) | Q9V3I8 |
| Code(thermo) | Q9YE60 |

| Name of protein | Aspartokinase 2 |
| --- | --- |
| Code(meso) | Q59229 |
| Code(thermo) | O58954 |

| Name of protein | 2-amino-5-formylamino-6-ribosylaminopyrimidin-4(3H)-one 5'-monophosphate deformylase |
| --- | --- |
| Code(meso) | D9PWM7 |
| Code(thermo) | Q8DIB4 |

| Name of protein | Peptidyl-tRNA hydrolase |
| --- | --- |
| Code(meso) | P0A7D1 |
| Code(thermo) | Q5L3U7 |

| Name of protein | Phosphate import ATP-binding protein |
| --- | --- |
| Code(meso) | P0AAH0 |
| Code(thermo) | Q5KX47 |

| Name of protein | Probable 3-phosphoshikimate 1-carboxyvinyltransferase |
| --- | --- |
| Code(meso) | B0R558 |
| Code(thermo) | Q9YEK9 |

| Name of protein | Probable cytosol aminopeptidase |
| --- | --- |
| Code(meso) | Q5H4N2 |
| Code(thermo) | Q9Y935 |

| Name of protein | Probable cytosol aminopeptidase |
| --- | --- |
| Code(meso) | Q65FE6 |
| Code(thermo) | Q8DI46 |

| Name of protein | Probable fructose-bisphosphate aldolase class 1 |
| --- | --- |
| Code(meso) | Q9Z8Q7 |
| Code(thermo) | Q9YG90 |

| Name of protein | Protein archease |
| --- | --- |
| Code(meso) | Q8PWN9 |
| Code(thermo) | Q9YFV2 |

| Name of protein | Protein archease |
| --- | --- |
| Code(meso) | Q9X0H1 |
| Code(thermo) | O59205 |

| Name of protein | Protein archease |
| --- | --- |
| Code(meso) | Q46D27 |
| Code(thermo) | Q9UX79 |

| Name of protein | Putative 1-aminocyclopropane-1-carboxylate deaminase |
| --- | --- |
| Code(meso) | Q32HE6 |
| Code(thermo) | O57809 |

| Name of protein | Putative 3-methyladenine DNA glycosylase |
| --- | --- |
| Code(meso) | Q65DF9 |
| Code(thermo) | Q9Y9P1 |

| Name of protein | Putative acetylglutamate kinase-like protein DR_1420 |
| --- | --- |
| Code(meso) | Q9RUG6 |
| Code(thermo) | O50147 |

| Name of protein | Ribulokinase |
| --- | --- |
| Code(meso) | Q9KBQ3 |
| Code(thermo) | B7GGV9 |

| Name of protein | ECOK1 Ribulokinase |
| --- | --- |
| Code(meso) | A1A7B0 |
| Code(thermo) | Q5KYP6 |

| Name of protein | Sodium/proton-dependent alanine carrier protein |
| --- | --- |
| Code(meso) | B9J358 |
| Code(thermo) | P30145 |

| Name of protein | Thermophilic beta-amylase |
| --- | --- |
| Code(meso) | F8FMD2 |
| Code(thermo) | P19584 |

| Name of protein | CARHZ Aldehyde ferredoxin oxidoreductase, tungsten-containing |
| --- | --- |
| Code(meso) | Q3ABF2 |
| Code(thermo) | O58778 |

| Name of protein | Acetylornithine/acetyl-lysine aminotransferase |
| --- | --- |
| Code(meso) | Q9RW75 |
| Code(thermo) | O59401 |

| Name of protein | NADP-dependent alcohol dehydrogenase 6 |
| --- | --- |
| Code(meso) | Q04894 |
| Code(thermo) | P14941 |

| Name of protein | N-glycosylase/DNA lyase |
| --- | --- |
| Code(meso) | Q9V3I8 |
| Code(thermo) | Q9YE60 |

| Name of protein | Aspartokinase 2 |
| --- | --- |
| Code(meso) | Q59229 |
| Code(thermo) | O58954 |

| Name of protein | 2-amino-5-formylamino-6-ribosylaminopyrimidin-4(3H)-one 5'-monophosphate deformylase |
| --- | --- |
| Code(meso) | D9PWM7 |
| Code(thermo) | Q8DIB4 |

| Name of protein | ECOLI Peptidyl-tRNA hydrolase |
| --- | --- |
| Code(meso) | P0A7D1 |
| Code(thermo) | Q5L3U7 |

| Name of protein | Phosphate import ATP-binding protein PstB |
| --- | --- |
| Code(meso) | P0AAH0 |
| Code(thermo) | Q5KX47 |

Table B. 50 Thermophilic proteins

| **SL.NO** | **PROTEIN NAME** | **UNIPROT ID** | **PDB ID** |
| --- | --- | --- | --- |
| 1 | 2,3-diketo-5-methylthiopentyl-1-phosphate enolase | Q5L1E2 | 2OEJ |
| 2 | 3-dehydroquinate dehydratase | Q5KY94 | 2YR1 |
| 3 | 30S ribosomal protein S28e | P61030 | 1NY4 |
| 4 | 50S ribosomal protein L12P | O57705 | 3A1Y |
| 5 | 282aa long hypothetical dehydrogenase | O59564 | 1FJH |
| 6 | Acyl carrier protein | Q5L0Q4 | 1F80 |
| 7 | Acylphosphatase | Q9YBK7 | 1W2I |
| 8 | Adenylosuccinate synthetase | O58187 | 2D7U |
| 9 | Asparagine--tRNA ligase | O57980 | 1X54 |
| 10 | Bifunctional protein | Q05213 | 3NGL |
| 11 | Cell division control protein 6 homolog | Q9YEV6 | 2VIU |
| 12 | Chromosome replication initiation protein | Q5KXY1 | 2VN2 |
| 13 | Citrate synthase | P21553 | 2lFC |
| 14 | DEAD-box ATP-dependent RNA helicase CshA | Q5L3G9 | 1HV8 |
| 15 | DNA polymerase | Q5KWC1 | 1NJW |
| 16 | DNA polymerase sliding clamp B | P57766 | 2HIK |
| 17 | Energy-coupling factor transporter ATP-binding protein EcfA 1 | Q5L3R0 | 1Z47 |
| 18 | Glyoxylate reductase | O58320 | 2DBQ |
| 19 | GTPase Der | Q5KXT0 | 1MKY |
| 20 | Holliday junction resolvase recU | Q5KXY4 | 2FCO |
| 21 | Conserved Hypothetical Protein | Q5KVJ9 | 2FCJ |
| 22 | Conserved Hypothetical Protein II | Q5L1S0 | 2BKM |
| 23 | Conserved Hypothetical Protein III | Q5KY38 | 1T0B |
| 24 | Hypothetical Conserved Protein IV | Q5QL47 | 2PCS |
| 25 | Hypothetical Conserved Protein V | Q5L106 | 2RSX |
| 26 | Hypothetical Conserved Protein VI | Q1CRX8 | 3VNP |
| 27 | Intracellular protease 1 | P10144 | 1G21 |
| 28 | Lysine--tRNA ligase | Q15046 | 1IRX |
| 29 | Malate dehydrogenase II | P40926 | 1GUZ |
| 30 | Malate Dehydrogenases | P0C890 | 1GV0 |
| 31 | Molybdenum cofactor biosynthesis protein | Q5L3F4 | 2EEY |
| 32 | N utilization substance protein B homolog | Q5KXA4 | 1EYV |
| 33 | N-acetyldiaminopimelate deacetylase | Q5L145 | 1XMB |
| 34 | NADH-quinone oxidoreductase subunit 1 | Q56222 | 4HE8 |
| 35 | Orotidine 5'-phosphate decarboxylase | Q5L0U0 | 2YYT |
| 36 | Peptide methionine sulfoxide reductase MsrA | Q65ID1 | 1FF3 |
| 37 | Pirin-like protein | Q5KZF0 | 2P17 |
| 38 | Probable galactokinase | O58107 | 2CZ9 |
| 39 | Protein-disulfide oxidoreductase | Q9YDZ4 | 2HLS |
| 40 | Putative uncharacterized protein | Q9Y8R5 | 3HA9 |
| 41 | Putative uncharacterized protein II | Q9YDN4 | 2PG4 |
| 42 | Ribonuclease P protein component 4 | O59248 | 1X0T |
| 43 | Ribulose bisphosphate carboxylase | O58677 | 2CWX |
| 44 | Sulfite reductase [NADPH] hemoprotein beta-component | Q5L041 | 7GEP |
| 45 | TATA-box-binding protein | Q9UWN7 | 1MP9 |
| 46 | Translation initiation factor 5A | O50089 | 1IZ6 |
| 47 | tRNA(Ile)-lysidine synthase | Q5L3T3 | 3A2K |
| 48 | V-type ATP synthase alpha chain | O57728 | 3I72 |
| 49 | V-type ATP synthase subunit F | P74903 | 2B00 |
| 50 | Xaa-Pro dipeptidase OS | O58885 | 1WY2 |

Table C. 50 Mesophilic proteins

| **SL.NO** | **PROTEIN NAME** | **UNIPROT ID** | **PDB ID** |
| --- | --- | --- | --- |
| 1 | 2,3-diketo-5-methylthiopentyl-1-phosphate enolase | O31666 | 2ZVI |
| 2 | 3-dehydroquinate dehydratase | Q48255 | 1J2Y |
| 3 | 30S ribosomal protein S28e | O26356 | 1NE3 |
| 4 | 50S ribosomal protein L12P | P29396 | 1DD3 |
| 5 | 282aa long hypothetical dehydrogenase | A5CYU4 | 2DHT |
| 6 | Acyl carrier protein | D3QU85 | 2QNW |
| 7 | Acylphosphatase | P07311 | 2ACY |
| 8 | Adenylosuccinate synthetase | Q9U8D3 | 1P9B |
| 9 | Asparagine--tRNA ligase | P58696 | 1LKH |
| 10 | Bifunctional protein | P24186 | 1B0A |
| 11 | Cell division control protein 6 homolog | Q99741 | 2CCH |
| 12 | Chromosome replication initiation protein | Q8XTV4 | 1V0G |
| 13 | Citrate synthase | P0ABH7 | 1K3P |
| 14 | DEAD-box ATP-dependent RNA helicase CshA | P96614 | 3G0H |
| 15 | DNA polymerase | P13382 | 3FLO |
| 16 | DNA polymerase sliding clamp B | P0A988 | 1JQL |
| 17 | Energy-coupling factor transporter ATP-binding protein EcfA 1 | P40735 | 3GFO |
| 18 | Glyoxylate reductase | Q9UBQ7 | 2CGG |
| 19 | GTPase Der | P0A6P5 | 2HJG |
| 20 | Holliday junction resolvase recU | P68818 | 2ZRN |
| 21 | Conserved Hypothetical Protein | O05815 | 2IB0 |
| 22 | Conserved Hypothetical Protein II | O07213 | 2WAM |
| 23 | Conserved Hypothetical Protein III | C1KXY4 | 3LPM |
| 24 | Hypothetical Conserved Protein IV | Q99YR1 | 1YHF |
| 25 | Hypothetical Conserved Protein V | Q8E972 | 1ZEE |
| 26 | Hypothetical Conserved Protein VI | Q5KW03 | 3HPE |
| 27 | Intracellular protease 1 | O59413 | 1FQ3 |
| 28 | Lysine--tRNA ligase | O57963 | 3BJU |
| 29 | Malate dehydrogenase II | P80039 | 2DFD |
| 30 | Malate Dehydrogenases | P61889 | 1GUY |
| 31 | Molybdenum cofactor biosynthesis protein | P65401 | 2Q5W |
| 32 | N utilization substance protein B homolog | B1XF04 | 1TZV |
| 33 | N-acetyldiaminopimelate deacetylase | A0RHZ2 | 2F7V |
| 34 | NADH-quinone oxidoreductase subunit 1 | C5W716 | 3RKO |
| 35 | Orotidine 5'-phosphate decarboxylase | P08244 | 1EIX |
| 36 | Peptide methionine sulfoxide reductase MsrA | D1YXW1 | 2IEM |
| 37 | Pirin-like protein | P42624 | 2VEC |
| 38 | Probable galactokinase | P51570 | 1WUU |
| 39 | Protein-disulfide oxidoreductase | Q12404 | 2ED3 |
| 40 | Putative uncharacterized protein | Q9WZW8 | 1WW1 |
| 41 | Putative uncharacterized protein II | Q9RS96 | 2YEU |
| 42 | Ribonuclease P protein component 4 | P34096 | 1RNF |
| 43 | Ribulose bisphosphate carboxylase | Q0INY7 | 1WDD |
| 44 | Sulfite reductase [NADPH] hemoprotein beta-component | P17846 | 1AOP |
| 45 | TATA-box-binding protein | P20226 | 1C9B |
| 46 | Translation initiation factor 5A | P63241 | 3CPF |
| 47 | tRNA(Ile)-lysidine synthase | Q3MIT2 | 2V9K |
| 48 | V-type ATP synthase alpha chain | P0ABB0 | 3GQB |
| 49 | V-type ATP synthase subunit F | P0ABA0 | 2OV6 |
| 50 | Xaa-Pro dipeptidase OS | P12955 | 2IW2 |
